# Evaluating barriers to sustainable boiler operation in the apparel manufacturing industry: Implications for mitigating operational hazards in the emerging economies

**DOI:** 10.1371/journal.pone.0284423

**Published:** 2023-04-14

**Authors:** Md. Tanvir Siraj, Binoy Debnath, Anil Kumar, A. B. M. Mainul Bari, Ashutosh Samadhiya, Spandan Basak Payel

**Affiliations:** 1 Department of Industrial and Production Engineering, Bangladesh University of Engineering and Technology, Dhaka, Bangladesh; 2 Guildhall School of Business and Law, London Metropolitan University, London, United Kingdom; 3 Jindal Global Business School, OP Jindal Global University, Sonipat, India; 4 Department of Mechanical Engineering, Bangladesh University of Engineering and Technology, Dhaka, Bangladesh; Libyan Academy, LIBYA

## Abstract

The efficiency with which conventional boilers perform, in terms of sustainability, is affected by a variety of factors. Unsustainable boiler operating practices are still surprisingly frequent in developing countries, resulting in environmental liabilities and catastrophic accidents. It is a serious problem in developing countries like Bangladesh, where boilers are utilized extensively in the apparel manufacturing sector. However, no research has yet examined the challenges or barriers associated with sustainable boiler operation in the apparel manufacturing sector. This study, thereby, utilizes an integrated MCDM approach, combining the fuzzy theory and the decision-making trial and evaluation laboratory (DEMATEL) method, to identify, prioritize, and explore the relations among the barriers to sustainable boiler operation in the apparel manufacturing industry, from an emerging economy perspective. The barriers were initially identified from the literature and a visual survey of 127 factories. After expert validation, thirteen barriers were finally selected to be analyzed utilizing the fuzzy DEMATEL method. The study findings revealed that ’Absence of water treatment facilities’, ’Fossil fuel burning and GHG emissions’, and ’Excessive consumption of groundwater’ are the three most prominent barriers to sustainable boiler operation. The cause-effect relations among the barriers suggest that ’Inadequate compliance with safety and hazard regulations’ is the most influential and ’Fossil fuel burning and GHG emissions’ is the most influenced barrier. This study is expected to guide the managers and policymakers of the apparel manufacturing sector in successfully overcoming the barriers to sustainable boiler operation, thus mitigating the operational hazards and achieving the sustainable development goals (SDGs).

## 1 Introduction

Industrial steam boilers are one of the essential components for the apparel manufacturing industries to produce steam for washing, drying, and ironing clothes [1]. Due to its indispensability in manufacturing, all the apparel industries install boilers in their production facilities. However, steam boilers consume a significant portion of energy and natural resource like water [2], and like most other heat-generating mechanical devices, it can negatively impact the environment, creating several criticalities to achieving sustainability [3, 4]. Global energy supply disruption due to unexpected situations like the COVID-19 pandemic or political upheavals like the Russia-Ukraine war is influencing policymakers to rethink and reconstruct conventional energy infrastructures [5, 6]. Like many other industries, the apparel manufacturing sector is also affected severely by these global energy crises, especially the countries with developing economies [7, 8]. Achieving sustainability in fuel-consuming utility machines like boiler operations in emerging economies can be groundbreaking in this context, effectively reducing the energy crisis and minimizing environmental impact.

The apparel manufacturing industry of Bangladesh is not only the highest revenue-earning sector for this emerging economic country but also one of the largest apparel manufacturing sectors in the world [9, 10]. This industry has contributed to around 85% of the country’s total export earnings in the last few decades and has established itself as a significant global player in this sector [11]. In this era of the fourth industrial revolution (Industry 4.0), to carry on with the achieved progress in the apparel sector, Bangladesh must broaden its focus to attain economic, social, and environmental sustainability in this sector. However, the apparel manufacturing industry heavily depends on steam boilers in their production processes. Hence, barriers to sustainable boiler operation can be considered a hindrance to achieving sustainability in this sector.

Over the last few years, researchers and practitioners have attempted to identify the factors of sustainable boiler operation in various ways. For instance, Rahman et al. [1] analyzed the causes of boiler accidents in the apparel industry to achieve workplace safety to ensure social and economic sustainability and identified non-standard boiler operation as one of the apparel industry’s main causative factors for boiler accidents. Bozzuto [12] described the critical success factors for sustainable boiler operations and emphasized skilled boiler operators for sustainable boiler operations. Moreover, Ranaraja et al. [13] explored the operating factors required to optimize the boiler’s performance and minimize energy consumption. Their study also focused on water quality improvement to get the optimum output from a boiler. Behzad et al. [14] tried to improve the sustainability of boiler operations by executing a maintenance strategy based on condition monitoring, periodic inspection, and predictive maintenance. Likewise, Patil et al. [15] proposed a reliability-centered maintenance approach to make the boiler operation economically sustainable. The study indicated that combined periodic and predictive maintenance might reduce a boiler’s operational cost. Kundakcı [16] applied a hybrid multi-criteria decision-making approach to finding the best boiler for sustainable operation and depicted steam generating cost as the most weighted criteria for boiler selection. Again, Li et al. [17] analyzed different fuel types in boiler operations to determine how they influence economic and environmental sustainability. Consequently, they showed the necessity of renewable energy to increase operational sustainability in boiler operations. Amid this situation, studying the barriers to sustainable boiler operation in the Bangladeshi apparel manufacturing industry context will convey valuable insights for all other emerging economies and thus can significantly contribute to achieving several sustainable development goals (SDGs).

Several recent research also explored different sustainability dimensions of boiler operations as follows: the safety factors of boiler operation to obtain social sustainability [18–20]; the fuel and other cost minimization strategies to obtain economic sustainability [21–23]; and the emission reduction and water consumption reduction strategies to obtain environmental sustainability [24, 25]. However, no study has considered achieving overall sustainability in boiler operation. Besides this, no previous work identified the barriers to sustainable boiler operation in the apparel manufacturing industry and explored cause-effect relationships among them to assist practitioners and decision-makers of emerging economies in mitigating these issues, which exposes a significant gap in the literature. Therefore, this study intends to fill this research gap by addressing the following research questions (RQs):

***RQ1***: What are the barriers that hinder the adoption of efficient practices in boiler operations to achieve sustainability in the apparel manufacturing industry?
***RQ3***: What are the most significant barriers to sustainable boiler operation in an emerging economy?
***RQ3***: How do the cause-effect interactions of the identified barriers influence each other in the context examined?

To fulfill the RQs mentioned above, this study outlines the following research objectives (ROs):

***RO1***: To identify the significant barriers to sustainable boiler operation in the apparel manufacturing industry.
***RO2***: To prioritize and categorize the identified barriers depending on their significance level in restricting the application of sustainable boiler operations.
***RO3***: To examine the correlations among the barriers and investigate their sustainability implications.

To accomplish the ROs, the barriers were initially determined by a systematic literature review, visual surveys, and factory inspection records. Later, these barriers were verified with the participation of relevant experts. Subsequently, the finalized barriers’ hierarchy and cause-effect linkages were evaluated using a combination of fuzzy set theory and the Decision-Making Trial and Evaluation Laboratory (DEMATEL) technique.

The conventional DEMATEL method can help prioritize and identify correlations amongst barriers, but it can not address the uncertainty and ambiguity in the expert’s feedback. Compared to the interpretive structural modeling (ISM) technique, DEMATEL enables an increased and broader assessment of underlying causes. For instance, whereas ISM only supports 0–1 interactions, DEMATEL allows bidirectional multi-number correlations [26]. However, a fuzzy-based decision-making framework can effectively minimize the uncertainty of human decision-making approaches [27, 28]. The key benefit of fuzzy DEMATEL is its ability to accommodate fuzziness and be agile while addressing uncertain instances [29]. In the fuzzy theory, analysts may quantify employing primal linguistics directly and using numerous membership function associations. The linguistic characterization is transformed into the analyst’s assessment score of the extent of the relationship or likelihood of the appearance of distinct phenomena [30].

Fuzzy DEMATEL is popular among researchers for providing prominence-based and cause-effect-based rankings [31]. This method has been used in several recent studies. For example, identifying cause-effect relationships among critical success factors for e-waste collection [32]; practices in a circular economy [33]; challenges to intelligent waste management [31]; barriers to sustainable food production [34] and so on. However, no previous study introduced the fuzzy DEMATEL methodology to analyze the barriers to the sustainable operation of any mechanical components used in any manufacturing industry, making this study unique.

The study considered exploring boiler operations of an emerging economic country like Bangladesh for two crucial reasons. First, emerging economies always have resource constraints compared while implementing sustainable practices, which can be expensive in many cases [35]. Second, the availability of methodically collected empirical data from industrial sectors is often limited in emerging economies like Bangladesh [1]. Considering this situation, a stratified strategy to mitigate the barriers to sustainable boiler operation one after another based on their prominence can be helpful for the decision-makers [27], and the analytical method of the study needs to utilize experts’ feedback due to the lack of availability of sufficient empirical data and directly relevant literature [36].

The rest of the paper is structured as follows: Section 2 conceptualizes the barriers to sustainable boiler operation in the apparel industry. Section 3 describes the methodology and calculations; Section 4 presents the results. Section 5 discusses the obtained results and the implications of the study. Finally, section 6 concludes the study and offers some future research scopes.

## 2 Conceptualizing the barriers to sustainable boiler operation

**Fig 1:**
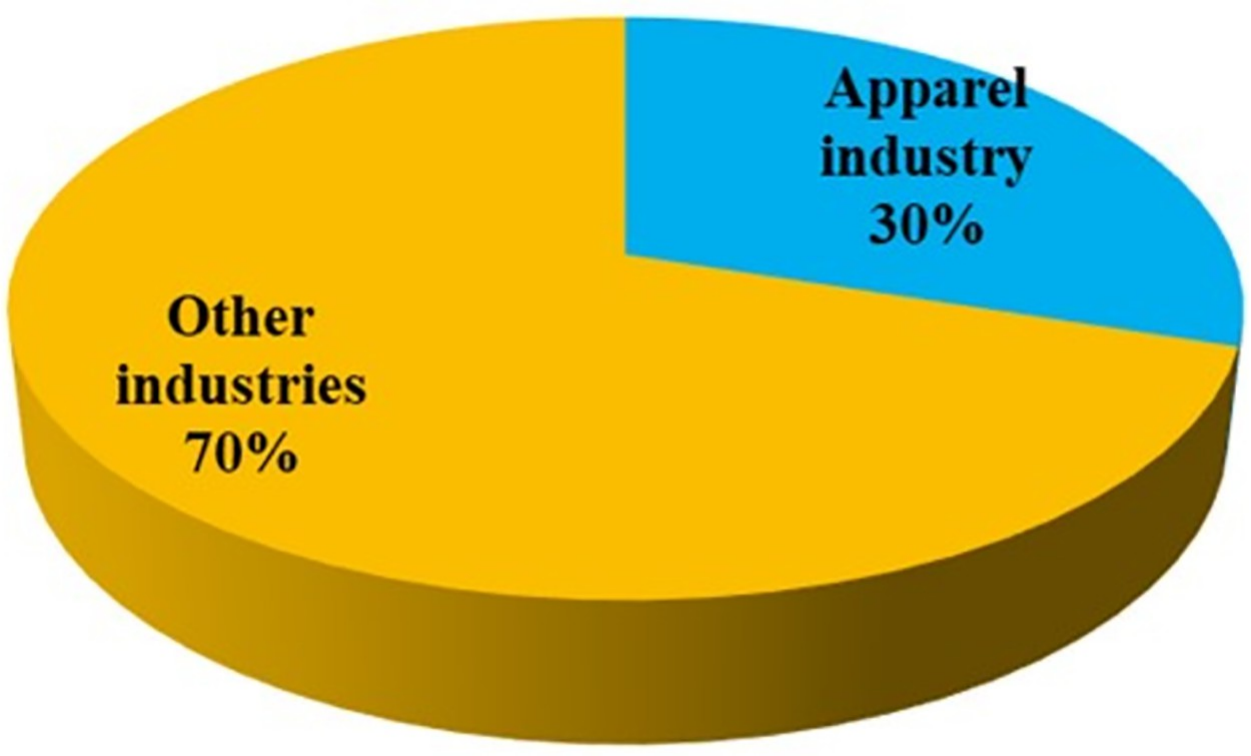
Approximate energy consumption scenario by industries in Bangladesh [38].

In Bangladesh, the apparel manufacturing industry occupies a significant portion of this country’s overall industrial energy consumption. Fig 1 shows the approximate energy consumption scenario of the apparel industry analyzed by the Bangladesh government’s Sustainable and Renewable Energy Development Authority (SREDA) in 2022. According to a recent webinar by the SREDA and German development agency GIZ, the apparel industry of Bangladesh consumes approximately 3,740 kilotons of oil equivalent (KTOE) of energy annually that has an opportunity to save 1,159 KTOE through sustainable operation [37].

Energy loss from the steam generation and distribution system in the apparel industry of Bangladesh is much higher than the international standard, which causes an increase in fuel consumption, directly affecting the production cost and environmental performance [2]. As boilers are a significant source of energy and water consumption in the apparel industry, ensuring sustainable boiler operations will drive the country’s overall industrial sustainability [21]. Moreover, workplace safety is a significant pillar of achieving sustainability which is also hampered by boiler accidents due to non-standard operational practices [1].

**Fig 2:**
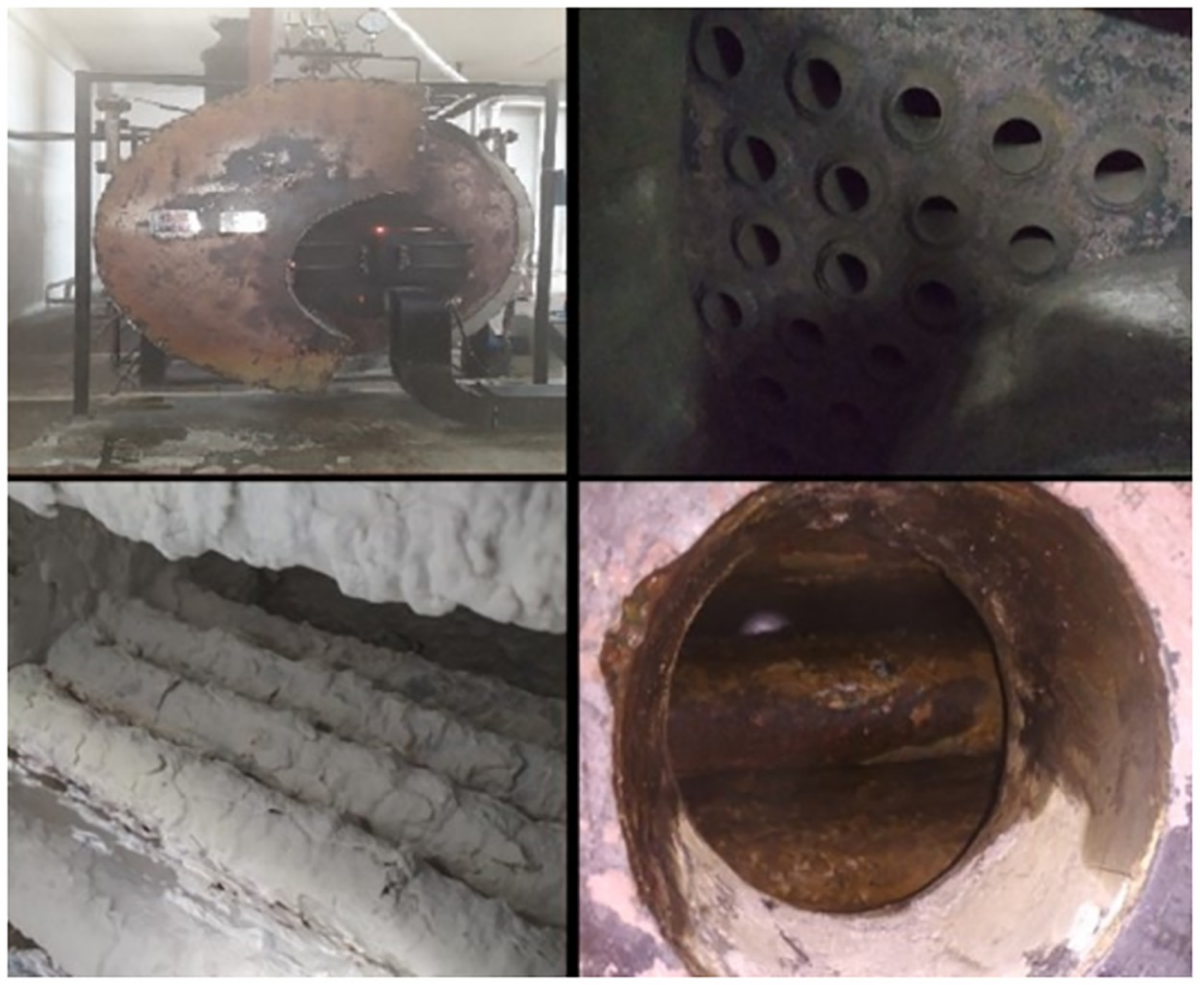
Heat dissipation from an uninsulated heated surface (Top-left), Rusted weld joint and carbon deposit due to unburnt fuel (Top-right), Corrosion of the boiler wall due to calcium build-up from untreated water (Bottom-left and Bottom-right) [39].

In the existing industrial structure of steam production and consumption, there are several barriers to achieving sustainability in boiler operation. The office of the chief inspector of boilers (http://www.boiler.gov.bd/) is the only overseeing body from the Bangladesh government that enforce legal boiler operation in the country following the Boiler Act-1923 and Bangladesh Boiler Regulations (BBR)-1951 [19]. There are detailed standardized guidelines for boiler operations in these legislations, such as—permitting only certified boilers, ensuring certified boiler operators, using softened feedwater for the boilers, conducting preventive and periodic maintenance, and so on. However, in industrial practice, the actual scenario is unsatisfactory, and there is a lot of scope for improvement to ensure sustainable boiler operation. Since 2018, the "Accord on Fire and Building Safety in Bangladesh", a non-government regulatory organization, which is now named simply as "ACCORD", has been conducting boiler inspections in the apparel industry. Those inspections have identified several barriers, such as corrosion of boiler metal due to untreated water, increase in fuel consumption due to scale formation, heat loss from uninsulated heating surface, water leakage, symptoms of unburnt fuel, and so on [39]. Fig 2 depicts some pictorial evidence of the barriers to sustainable boiler operation in the apparel industry found during boiler inspections by the ACCORD.

Several studies have also determined various barriers to sustainable boiler operation, which are relevant in the context of the Bangladeshi apparel industry. For instance, Hasan et al. [40] identified boiler explosions as a significant cause of industrial fire in apparel factories. A boiler explosion due to unsafe operation can cause fire and loss of life, hampering social and economic sustainability. Ali & Habibullah [19] reviewed the recent boiler accident case studies of the apparel industry and indicated that feeding direct groundwater to the boiler, lack of skilled workforce, and inadequate routine inspections are some of the primary reasons for unsustainable boiler operations. Paul & Alam [18] and Paul et al. [20] identified inadequate inspection facilities by the authority as one of the prime barriers to sustainable boiler operation in South Asian emerging economies. Hossan et al. [41] conducted a descriptive study and identified several causes and precautions for boiler explosions while emphasizing standard operations and maintenance practices to reduce boiler-related accidents. However, the abovementioned studies mainly focused on industrial safety and economic performance, not on social or environmental sustainability.

Moreover, Hasan et al. [42] discussed the impact of inefficient machinery on increasing fuel consumption in boiler operations. Old boilers generally lose their efficiency and consume more fuel which causes the emission of more carbon dioxide and other greenhouse gas (GHG), challenging environmental sustainability. Li et al. [17] suggested operating a boiler with renewable energy sources to reduce environmental hazards. However, Bangladesh’s renewable energy sources are not yet well-adopted [43]. Roushan [44] depicted that utilizing waste fabrics as a fuel instead of conventional fossil fuels can be cost-effective for boiler operation. However, the emission of greenhouse gas (GHG) is still a barrier to sustainability in such cases. Mamun et al. [9] and Haque et al. [45] denoted groundwater consumption without recycling it afterward as a barrier to economic and environmental sustainability. Therefore, blowdown is a mandatory operational process for a boiler where a vast amount of water is intentionally released after a time interval to improve the boiler’s performance. However, inadequate and improper recycling of blowdown water causes a lot of water wastage in the boiler operation. Besides, the natural groundwater level in Bangladesh is already declining. Hence, continuous pumping to lift this groundwater to be used in the boiler is imprudent and also responsible for extra energy consumption. Saha et al. [2] and Bukowska et al. [22] suggested that exhaust heat recovery systems such as water and air pre-heating or installing an economizer with a boiler can improve the cost-effective performance to a greater extent. An overview of the recent relevant studies has been presented in Table 1.

**Table 1:** Recent studies related to boiler operations.

Study	Objective	Applied Method	Outcomes
Rahman et al. [1]	Identifying and modeling the causes of boiler accidents	Analytic hierarchy process (AHP)	Developed a hierarchical model of the causes of boiler accidents in the apparel industry
Mamun et al. [9]	Finding a correlation between the amount of groundwater consumption and energy use in the apparel industry	Thermal energy calculations and descriptive statistics	Reducing the consumption of groundwater in the process can significantly reduce GHG emissions from fuel burning
Bose and Saini [4]	Determining the feasibility of an alternative energy source of fossil fuel for sustainable boiler operation	Descriptive statistics	Depicting renewable energy as a better energy source than fossil fuel to improve the environmental performance of boiler operation.
Saha et al. [2]	Analyzing the performance factors of the steam system in the apparel industry	Thermal energy calculations	Significant performance development can be achieved by heat recovery systems, thermal insulation, and some other standard practices
Haque et al. [45]	Determining a groundwater management policy for the apparel industry	Descriptive statistics and Regression analysis	Developing a sustainable industrial water projection tool for reducing groundwater consumption
Roushan [44]	Exploring an alternative to conventional fossil fuel for the boilers of the apparel industry	Descriptive statistics	Waste fabric can be a suitable, cost-effective alternative fuel for boiler operation
Paul et al. [20]	Exploring the safety practices in boiler operations	Descriptive statistics	Suggesting regular inspection and monitoring as a safety initiative against boiler accidents
Ranaraja et al. [13]	Identifying the factors for optimized boiler operation	Descriptive analysis	Discussing some of the most critical factors for boiler operation optimization
Hasan et al. [42]	Identifying the drivers and barriers to energy efficient apparel industry	Descriptive statistics	Ranking of the significant barriers and drivers
Ali and Habibullah [19]	Describing the existing legislation for boiler operation in Bangladesh	Descriptive analysis	Analyzing the previous boiler accidents and relating the causes with the existing standard practices
Hossan et al. [41]	Studying causes, consequences, and precautions for boiler accidents	Descriptive analysis	Suggesting some precautions by analyzing the causes and consequences of previous boiler explosions
Paul and Alam [18]	Analyzing compliance practices for boiler operations in South Asian countries	Descriptive statistics	Indicating Inadequate compliance with safety and hazard regulations as one of the significant causes of boiler accidents
Charde et al. [23]	Describing the performance-affecting factors for boiler operation	Descriptive analysis	Suggesting the development of some factors to increase the boiler’s efficiency
Bukowska et al. [22]	Analyzing the performance improvement of the boiler by introducing exhaust heat recovery systems	Thermal energy calculations and descriptive statistics	Showed the performance improvement after implementing the exhaust heat recovery systems from a case study
Li et al. [17]	Comparing the cost-effective performance of boiler operation by fossil fuel and renewable energy sources	Thermal energy calculations and descriptive statistics	Asserting the improvement of cost-effectiveness of renewable energy sources over fossil fuel

According to the above discussion, no study has attempted to identify the barriers to sustainability in boiler operations and find the causal relationships among those identified barriers to achieving SDGs in an emerging economy. Moreover, no study has established a contextual relationship among the discussed barriers either to assist the decision-makers. This demonstrates a significant gap in the relevant literature.

Several critical barriers to sustainable boiler operation could be conceptualized and accumulated from the existing studies and visual inspection reports, which can be analyzed in this research. Therefore, this study is expected to contribute to the prevailing literature in the following ways:

1. Highlighting the critical barriers to sustainable boiler operation in the apparel manufacturing industry of Bangladesh.
2. Developing a stratified ranking and categorization of the specified barriers to assist professionals and decision-makers in reducing the adverse impacts of these barriers.
3. Employing a fuzzy DEMATEL-based framework to assess the causal linkages among the barriers.
4. Conducting a sensitivity assessment to validate the viability of the presented methodology.
5. Guiding inspectors, authorities, and policymakers of emerging economies in strategically ensuring sustainable operations in the apparel manufacturing sector.

### 2.1 Identification of barriers

**Fig 3:**
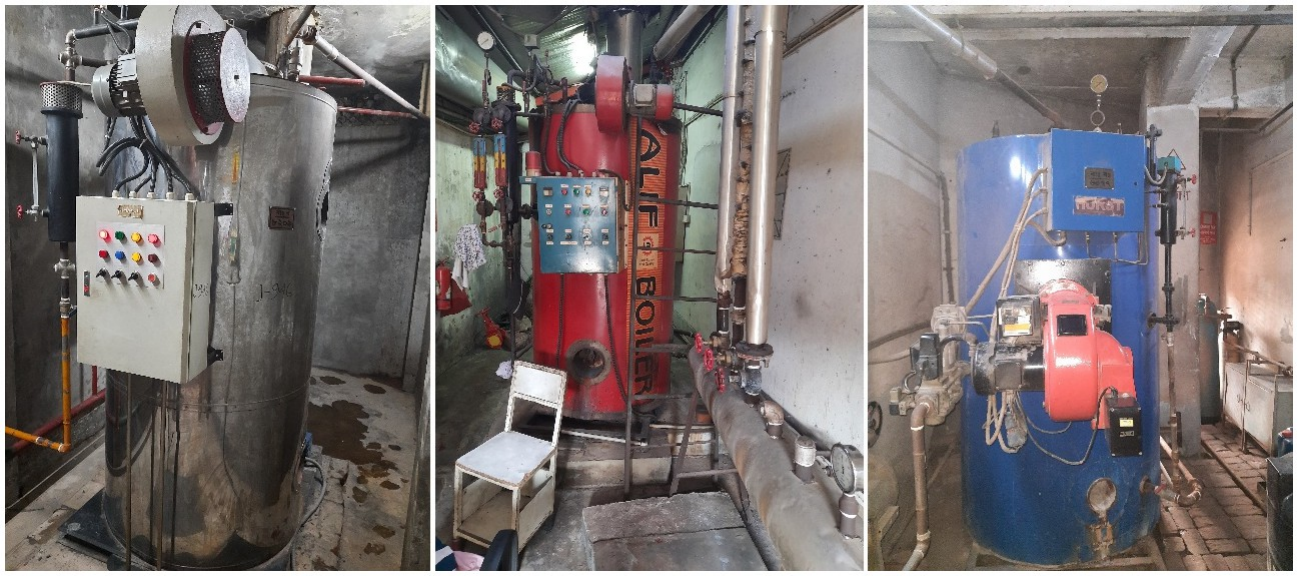
Improper ventilation for the boiler rooms observed during visual inspection.

For identifying the barriers in this study, besides reviewing the existing literature and analyzing the factory inspection report by the ACCORD [39], a visual survey in the boiler rooms of 127 apparel manufacturing factories situated at Mirpur, Tejgaon, and Savar industrial area in Dhaka Division, Bangladesh, has been conducted within a timeframe from March 2020 to August 2022. A team consisting of 6 mechanical engineers and two electrical engineers conducted this survey. This visual survey has validated multiple barriers to sustainable boiler operation, which were initially identified from the literature review. In addition, boiler rooms are found to be very congested, without proper ventilation facilities and other necessary safety features due to space constraints of the factories in the city areas. Improper ventilation and lack of oxygen flow in the boiler rooms can cause soot formation from unburnt fuel and increase fossil fuel consumption while operating the boiler [2]. Some evidence of congested boiler rooms observed during the visual survey is depicted in Fig 3.

Initially, a comprehensive literature analysis was carried out to identify the significant barriers to sustainable boiler operations in the apparel industry. The literature available in the Google Scholar and Sciencedirect database was reviewed for this purpose and was published between the years 2017 and 2023. Several key phrases were applied to search the barriers, such as: "Barriers to sustainable boiler operations," "Challenges to sustainability in boiler operation of the apparel industry," "Key obstacles to achieve sustainability in the garments industry of emerging economies," "Factors impeding the sustainability adoption in boiler operation in Bangladesh," and so on. The initial list of barriers to sustainable boiler operations identified from the literature search and visual inspection is provided in the S1 File as a questionnaire form. The form was subsequently distributed to the experts to validate the initially identified barriers.

## 3 Methodology and calculations

**Fig 4:**
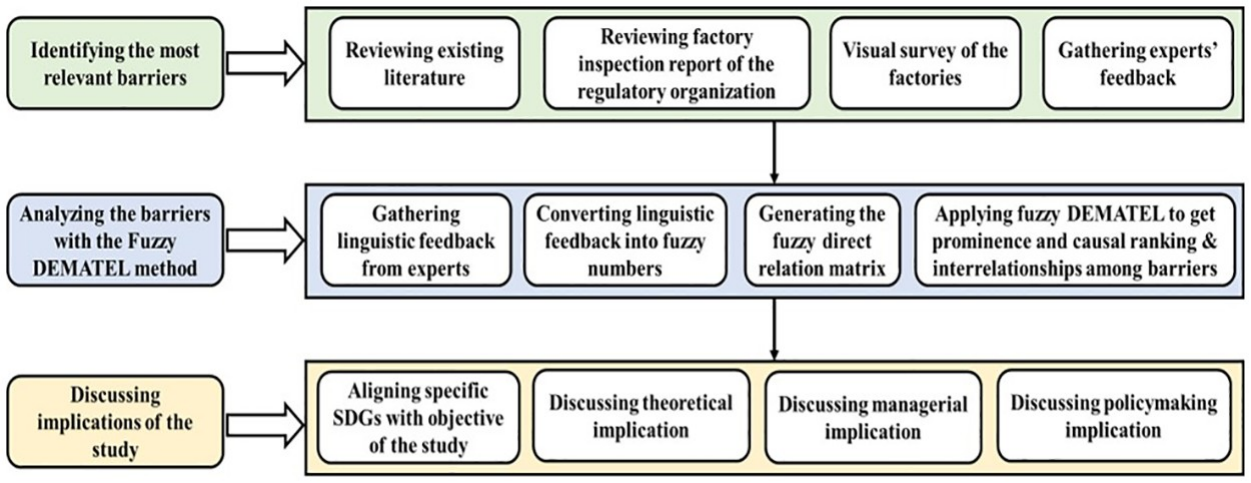
Proposed framework for this research.

This research was designed with three basic steps: identification of the barriers, analyzing the barriers using the fuzzy DEMATEL method, and discussing the study’s implications. Fig 4 shows the proposed research methodology.

### 3.1 Finalization of the barriers to sustainable boiler operations

For this research, a panel of 12 industrial experts was formed to gather feedback via a semi-structured questionnaire provided via Google Forms (see the S1 File). The list of barriers to be analyzed using the fuzzy DEMATEL method was finalized by utilizing the experts’ feedback. The experts were chosen using purposive sampling to utilize their expertise in this study [46, 47]. In such sampling of experts in decision-making studies, the number of experts may vary based on the researcher’s scope [48, 49]. Unlike other convenient sampling methods, purposive sampling requires a qualified and competent evaluation of the sample’s appropriateness and a compelling argument that a particular group of respondents offers a reliable foundation for study findings [50, 51]. The inclusion criteria for the experts were to have at least a bachelor’s degree in mechanical/electrical/environmental engineering, at least ten years of working experience in the apparel industry, a good understanding of the questionnaire, and a willingness to participate in the survey. Twenty experts were invited to validate the initially identified barriers, among which twelve responded (60% response rate). Later, those twelve experts were invited again to evaluate barriers using the fuzzy DEMATEL method, and all of the twelve invited experts responded in this stage of feedback collection (100% response rate). A summary of the twelve participating experts’ profiles is presented in Table 2.

**Table 2:** Profile of the experts.

Experts	Education	Designation	Experience
Expert 1	MSc in Electrical Engineering	Head of Operations	20 years
Expert 2	BSc in Electrical Engineering	General Manager	17 years
Expert 3	MSc in Mechanical Engineering	Compliance Manager	12 years
Expert 4	MSc in Environmental Engineering	Compliance Manager	10 years
Expert 5	BSc in Mechanical Engineering	Maintenance Manager	14 years
Expert 6	BSc in Electrical Engineering	Production Manager	13 years
Expert 7	BSc in Environmental Engineering	Health and Safety Manager	12 years
Expert 8	BSc in Electrical Engineering	Compliance Engineer	10 years
Expert 9	BSc in Mechanical Engineering	Operations Manager	11 years
Expert 10	MSc in Mechanical Engineering	Utility Manager	18 years
Expert 11	MSc in Environmental Engineering	Head of Workplace Safety	16 years
Expert 12	BSc in Environmental Engineering	Industrial Safety Manager	12 years

Initially, 13 critical barriers to sustainable boiler operation were identified (see the S1 File) from the literature review and the visual inspection report, which was later sent to the experts for validation, whether the identified barriers are relevant and significant to the context examined. Experts removed one barrier (Lack of automation) from the initial list and added one more barrier ("Inadequate supply and availability of spare parts"). According to the experts, most industrial boilers, boiler-related accessories, and water treatment systems operated in the Bangladeshi apparel industry are imported from foreign countries, which often leads to the inadequate availability of various necessary spare parts. Thus, 13 barriers were finally selected to be evaluated by using the fuzzy DEMATEL method, which is presented in Table 3. A concise description of these finalized barriers is provided in Table S1 of the S1 File.

**Table 3:** Identified barriers to sustainable boiler operation.

ID	Barrier	Source
B1	Usage of old and inefficient infrastructure	Hasan et al. [42]
B2	High dependency on nonrenewable energy sources	Li et al. [17]; Anam et al. [43]
B3	Fossil fuel burning and GHG emissions	Roushan [44]; Barma et al. [21]
B4	Improper heat insulation	ACCORD [39]; Saha et al. [2]
B5	Absence of water treatment facilities	Ranaraja et al. [13]; Bozzuto [12]
B6	Lack of periodic inspection and preventive maintenance	Alazemi et al. [52]; Behzad et al. [14]; Patil et al. [15]
B7	Absence of heat recovery system	Charde et al. [23]; Fialko et al. [25]
B8	Not recycling blowdown water	Haque et al. [45]; Barma et al. [21]
B9	Excessive consumption of groundwater	Ali and Habibullah [19]; Haque et al. [45]
B10	Lack of skilled manpower	Paul et al. [20]; Rahman et al. [1]
B11	Inadequate compliance with safety and hazard regulations	Paul & Alam [18]; Ali and Habibullah [19]
B12	Inadequate boiler room ventilation and safety measures	Visual survey
B13	Inadequate supply and availability of spare parts	Expert’s feedback

### 3.2 Fuzzy DEMATEL method

The DEMATEL method is most commonly utilized to understand the structure of complex causal relationships through digraphs, and it has become very popular due to its simplicity of interpretation [53, 54]. The fuzzy set theory is often coupled with the conventional DEMATEL method to improve its responsiveness towards ambiguity arising from human judgment or expert feedback [55]. The fuzzy set theory has been discussed briefly in the S1 File. This study utilized a fuzzy DEMATEL-based framework to illustrate the cause-effect correlations among barriers and to overcome perceived ambiguity and uncertainty. Details procedure of the fuzzy-based DEMATEL framework that was applied in this study [32], has been discussed in the S1 File.

## 4 Results

The step-by-step calculations using the fuzzy DEMATEL method have been presented in the S1 File. In the S1 File, Table S2 shows the linguistic scale used to collect the experts’ feedback, Table S3 shows the aggregated direct-relationship matrix, Table S4 shows the de-fuzzified crisp direct-relation matrix, Table S5 shows the normalized crisp relation matrix, and Table S6 presents the total relation matrix “*T*”.

The prominence ranking of the barriers to sustainable boiler operation in the apparel manufacturing industry is shown in Table 4. The relative weights (RW) of the barriers can improve the visibility of the determined prioritization and depict the relative significance of one barrier over another.

**Table 4:** Prominence ranking of the barriers to sustainable boiler operation.

Denotation	Barrier	D+R	RW	Ranking
B5	Absence of water treatment facilities	1.683	0.1331	1
B3	Fossil fuel burning and GHG emissions	1.580	0.1250	2
B9	Excessive consumption of groundwater	1.489	0.1178	3
B11	Inadequate compliance with safety and hazard regulations	1.375	0.1088	4
B8	Not recycling blowdown water	1.193	0.0944	5
B6	Lack of periodic inspection and preventive maintenance	1.083	0.0856	6
B10	Lack of skilled manpower	0.974	0.0770	7
B13	Inadequate supply and availability of spare parts	0.960	0.0760	8
B1	Usage of old and inefficient infrastructure	0.930	0.0736	9
B2	High dependency on nonrenewable energy sources	0.457	0.0361	10
B4	Improper heat insulation	0.391	0.0309	11
B7	Absence of heat recovery system	0.266	0.0210	12
B12	Inadequate boiler room ventilation and safety measures	0.261	0.0207	13

Here, all the RWs are summed up to 1. The ranking of barriers is determined in descending order of the influence level (D+R) of each barrier. Hence, the ranking of barriers obtained in this study is: B5 > B3 > B9 > B11 > B8 > B6 > B10 > B13 > B1 > B2 > B4 > B7 > B12.

The causal ranking of the barriers is presented in Table 5. In that ranking, the barriers are divided into two groups, the cause group, and the effect group. Six barriers were found as causal barriers (where D-R > 0), and seven barriers were found as effect barriers (where D-R < 0). The barriers in the cause group are ranked as: B11 > B6 > B13 > B2 > B12 > B7. The barriers in the effect group are ranked as: B5 > B10 > B8 > B1 > B4 > B9 > B3.

**Table 5:** Causal ranking of the barriers (the cause group and the effect group).

Denotation	Barrier	D-R	Ranking	Group
**B11**	Inadequate compliance with safety and hazard regulations	1.3749	1	**Cause Group**
**B6**	Lack of periodic inspection and preventive maintenance	0.7963	2	
**B13**	Inadequate supply and availability of spare parts	0.4047	3	
**B2**	High dependency on nonrenewable energy sources	0.2522	4	
**B12**	Inadequate boiler room ventilation and safety measures	0.1660	5	
**B7**	Absence of heat recovery system	0.0158	6	
**B5**	Absence of water treatment facilities	-0.0643	1	**Effect Group**
**B10**	Lack of skilled manpower	-0.0994	2	
**B8**	Not recycling blowdown water	-0.1227	3	
**B1**	Usage of old and inefficient infrastructure	-0.1910	4	
**B4**	Improper heat insulation	-0.2974	5	
**B9**	Excessive consumption of groundwater	-0.9084	6	
**B3**	Fossil fuel burning and GHG emissions	-1.3269	7	

**Fig 5:**
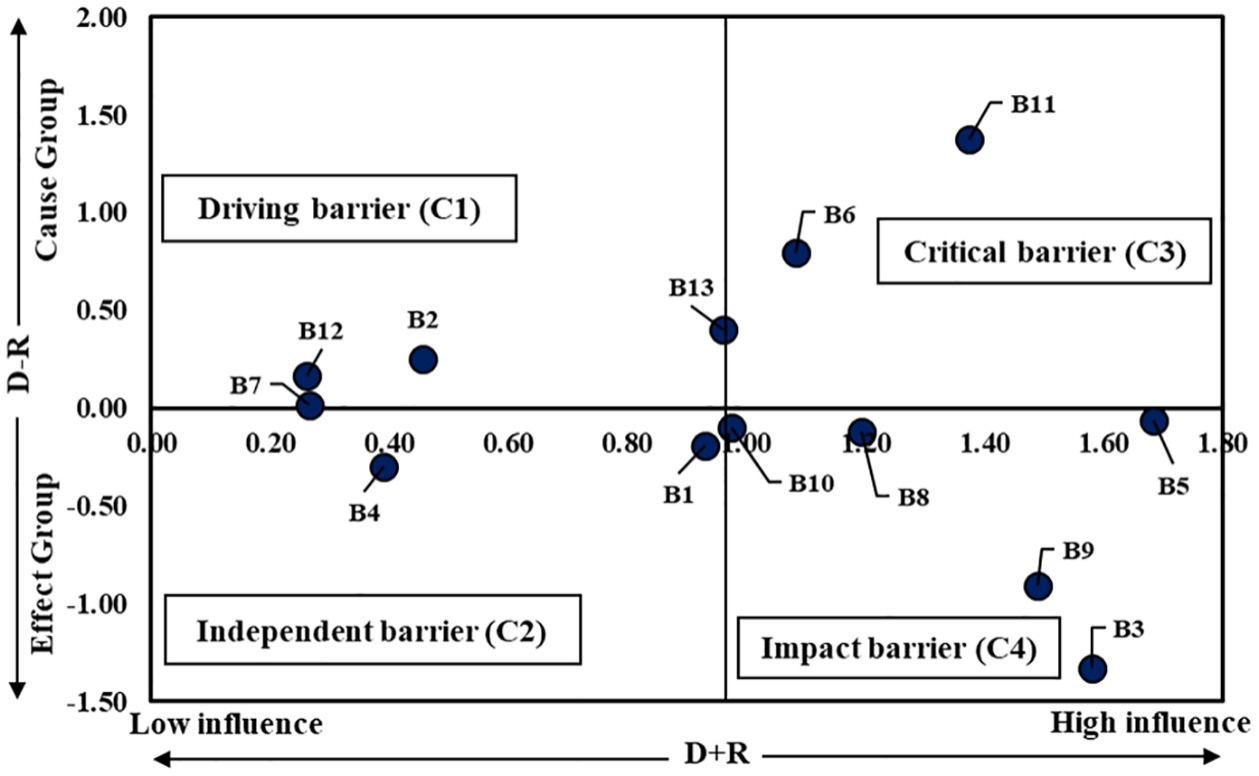
Representation of the cause and effect classification of barriers.

Fig 5 presents a causal diagram generated using Tables 4 and 5. The diagram was drawn on the Cartesian Plane with D+R as X-axis and D-R as Y-axis. The barriers are clustered into four categories, driving category (C1), independent category (C2), critical category (C3), and impact category (C4). The driving barriers and critical barriers represent the cause group in these categories. On the other hand, the independent barriers and the impact barriers represent the effect group. C3 depicts the most influencing barriers as this category lies in the cause group and the high influence region. Policymakers must emphasize diminishing these barriers to mitigate the unsustainable practices of boiler operation in the apparel industry. On the other hand, C4 depicts the most affected barriers placed in the effect group.

**Fig 6:**
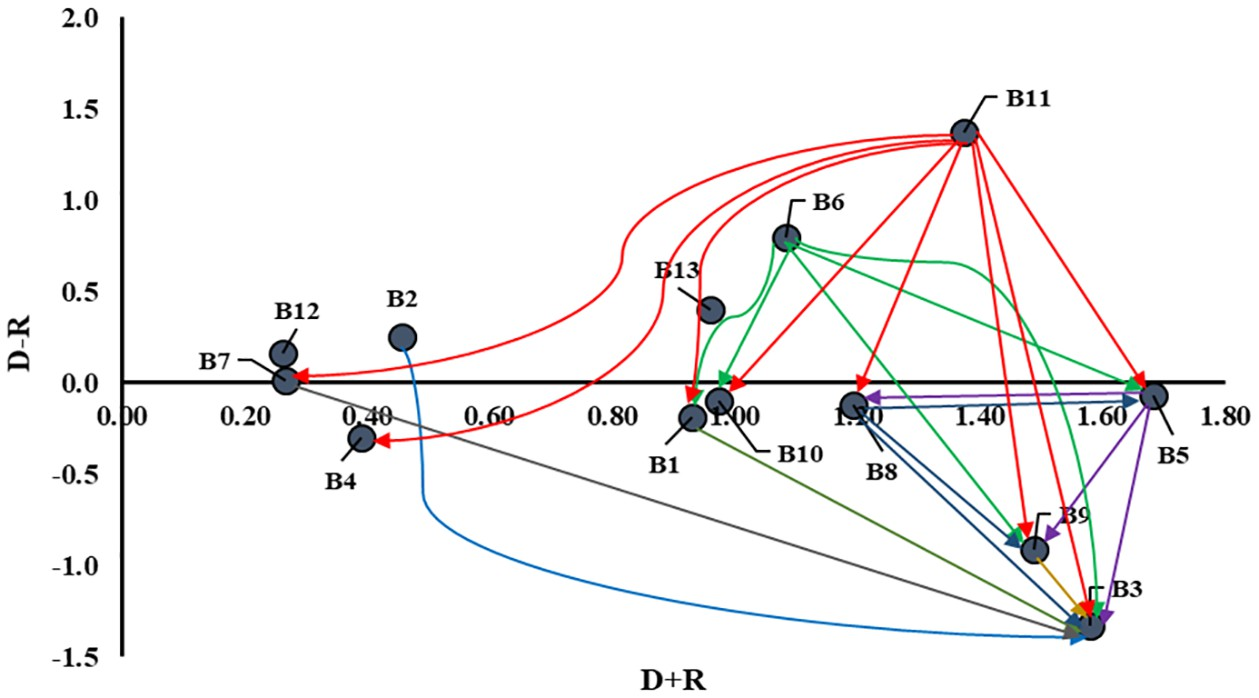
Relationships among the barriers.

The causal relationships among the barriers are presented in Fig 6. For 13 barriers, the maximum possible number of relationships is 13 × 13. Depicting this large number of relationships in a single diagram makes it visually complicated. Therefore, this study set a threshold value (**θ** = 0.0957) using equation (11) (see the S1 File).

### 4.1 Sensitivity analysis to check the robustness of the findings

Sensitivity analysis is a necessary experimental approach to checking the stability and robustness of a decision-making study [56]. There are several techniques to analyze the sensitivity of a study result [36, 57, 58]. This study followed the technique Akter et al. [36] described for analyzing the obtained result’s stability.

**Fig 7:**
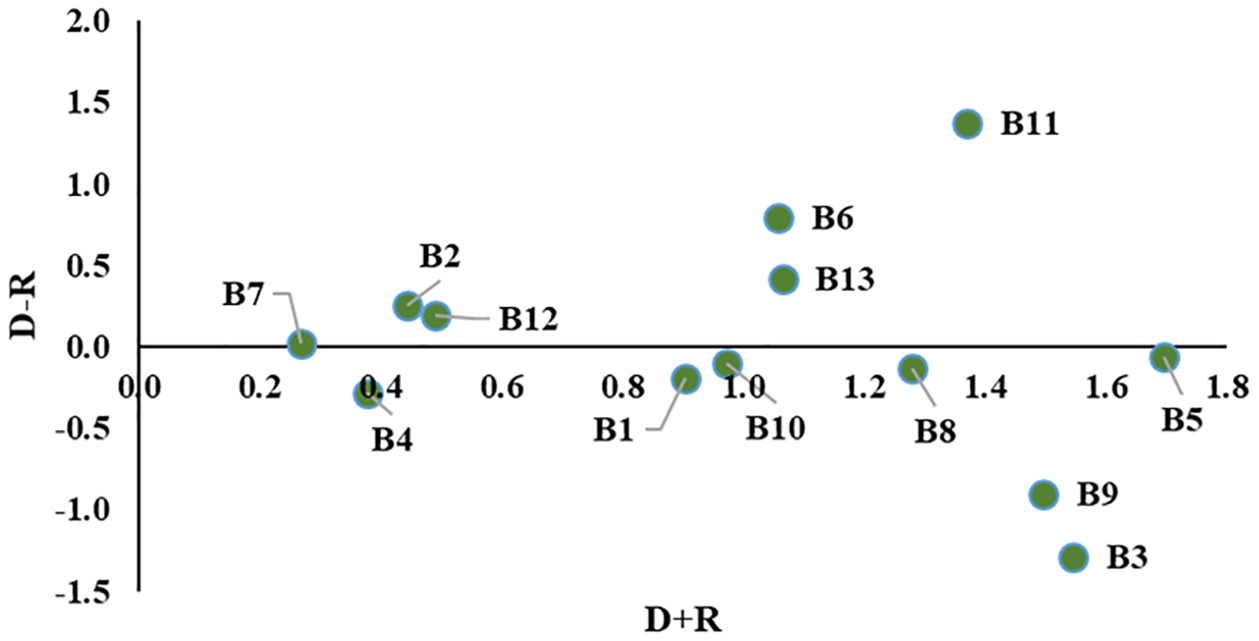
The relationships among the barriers for sensitivity analysis Case 1.

**Fig 8:**
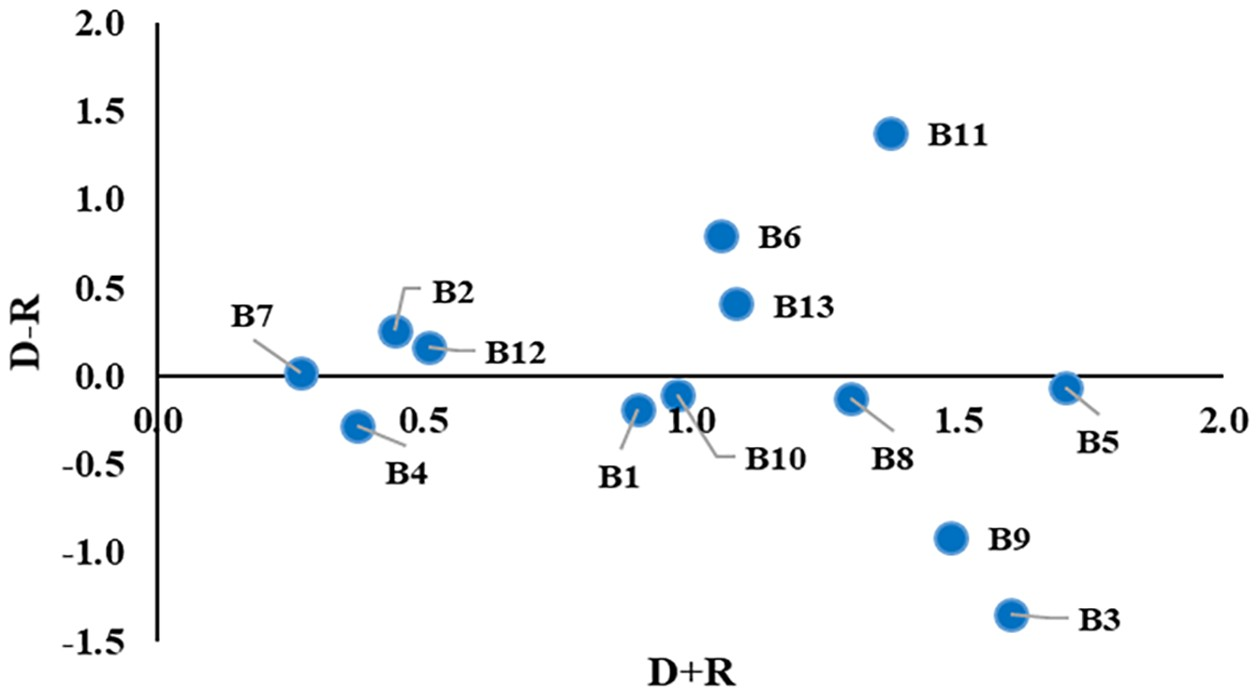
The relationships among the barriers for sensitivity analysis Case 2.

**Fig 9:**
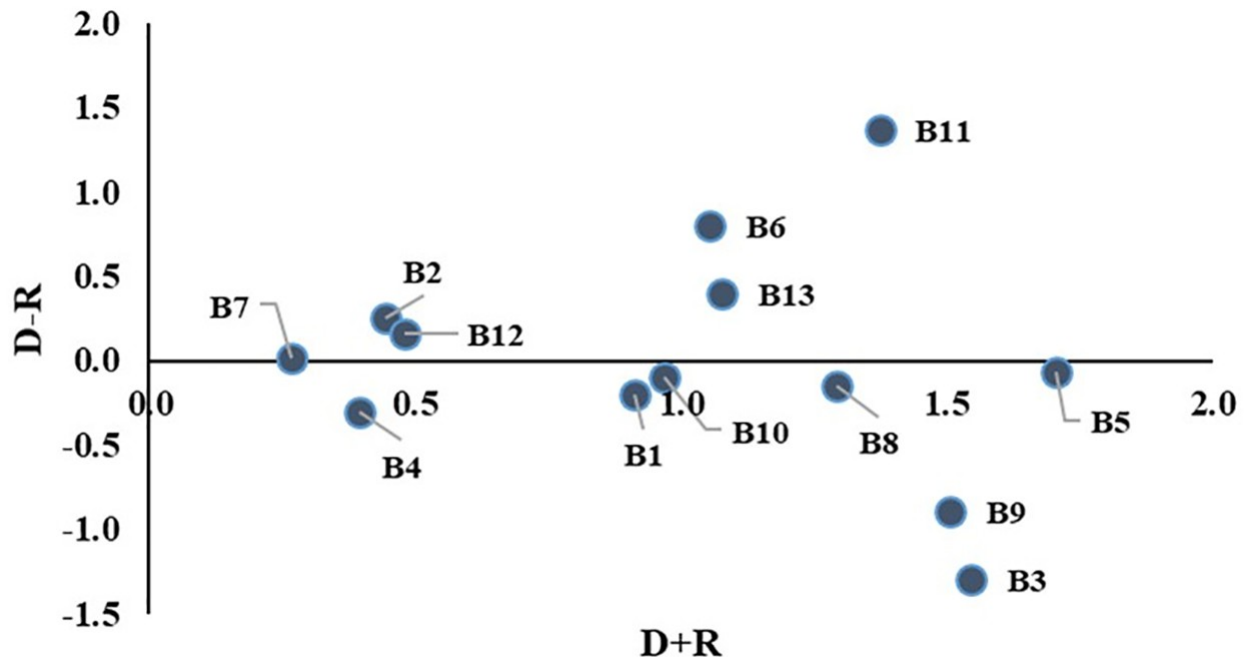
The relationships among the barriers for sensitivity analysis Case 3.

Initially, all the experts’ evaluation scores were equally weighted in this study. However, the educational backgrounds of the experts were different, which might create bias-ness and minimize the results’ credibility. Hence, this study imposed different weights on different educational backgrounds and created three case scenarios with similar evaluation scores from the experts. The three case scenarios of the sensitivity analysis by assigning different weights can be found in Table 6. The outcomes of the sensitivity analysis are presented in Figs 7–9.

**Table 6:** Assigned weights based on the educational background of the experts.

Case scenario	Mechanical Engineer	Electrical Engineer	Environmental Engineer
Case-1	0.4	0.3	0.3
Case-2	0.3	0.4	0.3
Case-3	0.3	0.3	0.4

**Fig 10:**
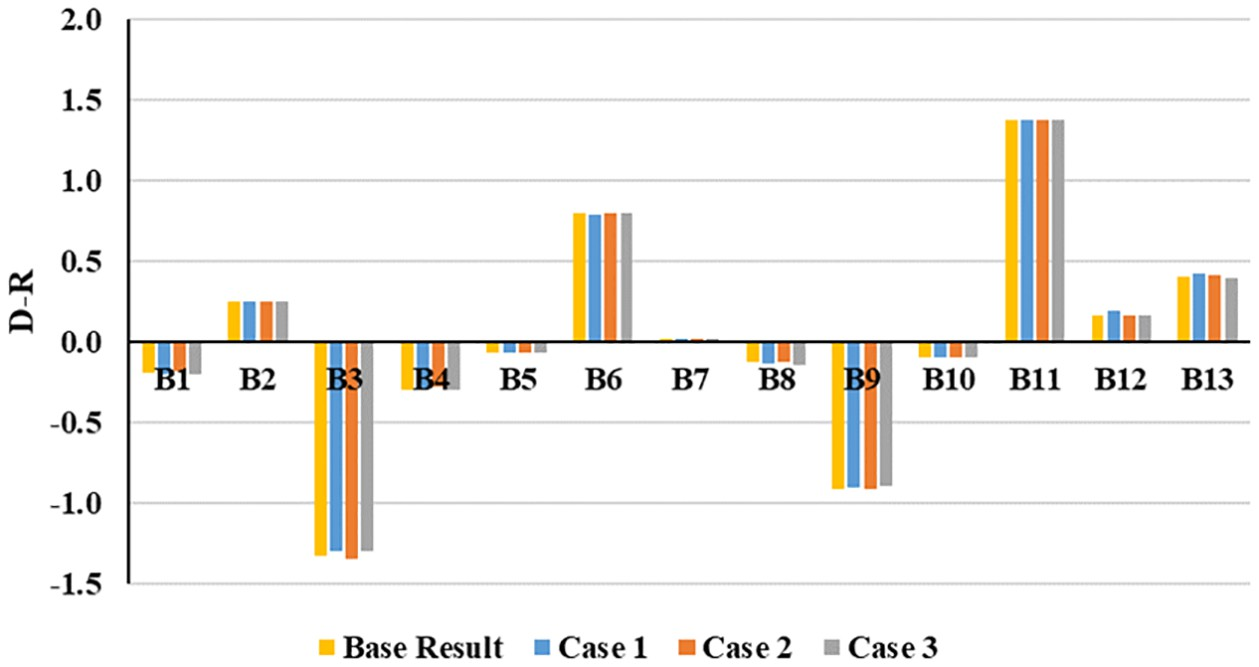
Comparison of different cases and base result.

Table 7 and Fig 10 compare the three case scenarios with the base result obtained in this study.

**Table 7:** Comparison among different cases of sensitivity analysis and the base result.

Barrier ID	D-R values			
	Base result	Case 1	Case 2	Case 3
**B1**	-0.191	-0.197	-0.185	-0.197
**B2**	0.252	0.252	0.254	0.253
**B3**	-1.327	-1.292	-1.346	-1.293
**B4**	-0.297	-0.285	-0.282	-0.301
**B5**	-0.064	-0.062	-0.064	-0.062
**B6**	0.796	0.793	0.794	0.800
**B7**	0.016	0.016	0.016	0.016
**B8**	-0.123	-0.134	-0.123	-0.146
**B9**	-0.908	-0.900	-0.913	-0.893
**B10**	-0.099	-0.097	-0.100	-0.094
**B11**	1.375	1.371	1.376	1.374
**B12**	0.166	0.194	0.166	0.163
**B13**	0.405	0.420	0.410	0.398

The sensitivity analysis shows very little variation in the causal values of the variables (see Fig 10 and Table 7), and their order within the group remained consistent across all scenarios and the base results. This consistency indicates the stability and robustness of the obtained result.

## 5 Discussions

According to the prominence ranking shown in Table 4, ’Absence of water treatment facilities (B5)’ carries the most significant among all the barriers to sustainable boiler operation. Many apparel manufacturing factories are not concerned about the quality of feedwater used for the boilers, which damages the physical condition of the boilers and is a major reason for many environmental and safety hazards in boiler operation [59, 60]. A boiler’s lifespan is negatively impacted by the presence of natural groundwater due to the presence of harmful substances, such as calcium and magnesium salts and iron ions, which cause metal degradation and scale development. This sort of internal damage might cause the boiler to lose efficiency or perhaps explode. Therefore, it is imperative for textile mills to prioritize the installation of water treatment facilities for boilers in order to reduce risks and maintain the long-term sustainability of boiler operations.

The second most significant barrier to sustainable boiler operation appears to be the ’Fossil fuel burning and GHG emissions (B3)’. Boilers in the apparel industries of emerging economies mostly burn fossil fuels to generate steam [44]. Part of the SDGs to safeguard the environment is to reduce the use of fossil fuels and the release of GHG. The boiler is one of the most fuel-intensive machinery components in the apparel manufacturing operation; this barrier has a detrimental effect on boiler sustainability, and by extension, on overall sustainability. As a result, we need to figure out how to use renewable energy sources instead of fossil fuels in more applications.

The third most important barrier is the ’Excessive consumption of groundwater (B9)’. Overexploting of groundwater has become a crucial challenge to attaining sustainability in many countries of the world [61]. Consuming excessive groundwater in industrial manufacturing destroys the ecosystem [62]. Effective measures, such as using recycled industrial water and water from natural water bodies like lakes or rivers after adequate treatment, can minimize the quantity of groundwater used by steam boilers in apparel manufacturing companies [2]. The apparel sector may boost its sustainability performance with the aid of these strategies.

The fourth most crucial barrier determined in this study is ’Inadequate compliance with safety and hazard regulations (B11)’. The practice of conducting regular inspections of the boilers to ensure safety and regulatory compliance is not very common in emerging economies due to various technical, financial, and socio-economic constraints [20, 41, 63]. But, developed countries always ensure regular inspections by different government and non-government authorities to establish standard operational practices, thus minimizing environmental and safety hazards [64, 65]. Therefore, government and industry owners of emerging economies should strive more to ensure compliance with the existing safety and hazard regulations to ensure sustainable boiler operations in the apparel industry.

’Not recycling blowdown water (B8)’ is the fifth most significant barrier to sustainable boiler operation. Boiler operation necessitates periodic "blowdown", in which part of the boiler’s water is drained and replaced with freshwater in order to keep the boiler water quality within the allowed range. In order to improve thermal efficiency and decrease the amount of dissolved particles in the boiler water, blowdown is an essential stage in boiler operation. However, a blowdown results in the loss of thermal energy and water [66, 67]. Moreover, releasing boiler water directly into the environment can cause chemical toxicity, which can be hazardous to the ecosystem [68]. Therefore, recycling blowdown water can improve energy efficiency and save the ecosystem. This way, recycling the blowdown water can enhance sustainability.

According to Table 5 and Fig 5, ’Inadequate compliance with safety and hazard regulations (B11)’ is observed to be the most impactful and influencing on other barriers among all causal barriers. This indicates following standardized rules and regulations can contribute to sustainable operation. Inadequate or no inspection can result in various non-compliance [65]. In such a situation, a highly sophisticated device like the boiler must be inspected regularly to ensure compliance with all the standards and safety practices [1]. Removing this causal barrier can directly influence removing other significant barriers to sustainable boiler operation.

’Lack of periodic inspection and preventive maintenance (B6)’ is discovered to be the second-ranked casual barrier. This barrier degrades the quality of regular operations and maintenance of the boiler. To find any fault instantly, boiler operators should regularly inspect the boiler room, fuel lines, and steam lines, reducing steam and energy loss and minimizing any safety hazards [69, 70]. Again, preventive maintenance is a part of maintenance activity that can increase the reliability of a boiler to a great extent [52, 71]. Hence, the practice of preventive maintenance should be adopted in the industry to ensure the safety and sustainability of boiler operations.

’Inadequate supply and availability of spare parts (B13)’ was found to be the third most significant causal barrier in this study. Inadequate supply and availability of spare parts cause import dependency on foreign countries for industrial components. This can directly hamper the routine maintenance of the boiler and thus can reduce its operational reliability and sustainability [72, 73].

’High dependency on nonrenewable energy sources (B2)’, ’Inadequate boiler room ventilation and safety measures (B12)’, and ’Absence of heat recovery system (B7)’ are the following three barriers from the causal ranking, respectively. The high dependency on nonrenewable energy sources is one of the significant barriers to achieving SDGs [43]. The absence of renewable fuels causes many unsustainable practices, including fossil fuel burning. In addition, most Bangladeshi apparel manufacturing factories are installed in very congested areas, for which boiler room ventilation and other safety measures are often inadequate. Proper ventilation can also reduce fuel consumption and GHG emissions [74]. Again, without proper ventilation, operators’ comfort in the boiler room is greatly hampered by the exceeding temperature, which is a significant safety concern. Besides these, heat recovery systems like preheaters, economizers, condensers, etc., are not usually utilized with boilers in the apparel manufacturing sector of emerging economies like Bangladesh. The absence of heat recovery systems can lead to many other problems, like increased fossil fuel consumption or increased GHG emission, which can negatively impact operational sustainability [75].

The seven effect barriers determined in this study (B5 > B10 > B8 > B1 > B4 > B9 > B3) are directly influenced by the six causal barriers. Barriers like ’Absence of water treatment facilities (B5)’ and ’Lack of skilled manpower (B10)’ are less impacted than other effect group barriers; instead, they are vital due to lying near the causal barriers. However, they are still influenced by the cause group barriers. For instance, ’Inadequate compliance with safety and hazard regulations (B11)’ can result in the ’absence of water treatment facilities (B5)’. In the standardized guidelines, installing a water treatment facility is mandatory to operate a boiler [19]. ’Inadequate supply and availability of spare parts (B13)’ also influences the ’Absence of water treatment facilities (B5)’, since the unavailability of components may discourage the management from setting up a new water treatment facility. Besides this, the ’Lack of skilled manpower (B10)’ is impacted by the ’Lack of periodic inspection and preventive maintenance (B6)’ and ’Inadequate compliance with safety and hazard regulations (B11)’. Periodic inspections and regular preventive maintenance can develop the operational, maintenance, and troubleshooting skills of the existing human resources of the industry. Moreover, industry owners will emphasize developing a skilled workforce if there is regular monitoring from legislative and law enforcement authorities.

’Not recycling blowdown water (B8)’ is strongly influenced by the casual barriers’ Inadequate compliance with safety and hazard regulations (B11)’ and ’Inadequate supply and availability of spare parts (B13)’. Interestingly, an effect barrier, ’absence of water treatment facilities (B5)’, also strongly influences that barrier. Recycling blowdown water is often considered a waste of money since it will require imported foreign components, which is a stereotype. Studies confirm that blowdown water recycling not only helps to save natural resources but also improves the economic performance of boiler operations [66, 67]. Factories often cannot recycle the blowdown water due to the absence of water treatment facilities.

Barriers like ’Usage of old and inefficient infrastructure (B1)’, ’Improper heat insulation (B4)’, ’Excessive consumption of groundwater (B9)’, and ’Fossil fuel burning and GHG emissions (B3)’ are strongly influenced by various causal barriers and some of the effect barriers. Therefore, these barriers were ranked last in the causal ranking in this study.

Fig 6 of this study depicts that ’Inadequate compliance with safety and hazard regulations (B11)’ has the most significant causal relationships with other barriers, and the causal barrier ’Lack of periodic inspection and preventive maintenance (B6)’ is also strongly related to other barriers. Therefore, management and policymakers should first emphasize removing these two barriers to facilitate sustainability attainment in boiler operations.

For the sensitivity analysis, experts’ experience level, job responsibilities, or educational level can be considered for imposing different weights [76]. This study considered the educational background of the experts while performing the sensitivity analysis. Clustering was conducted based on the same educational background (see Table 6 and Figs 7 to 9). A few minor variations were observed for different cases, which indicates the stability and robustness of the obtained results (see Table 7 and Fig 10).

The findings obtained from this study are significantly different from the other relevant studies. For instance, Rahman et al. [1] utilized the AHP method to explore the causes of boiler explosions in the apparel industry, which found a lack of standard legislation and non-standard boiler operation as the most critical causes of boiler accidents. Paul et al. [20] utilized descriptive statistics to identify the significant reasons for boiler explosions in developing South Asian countries. They identified non-compliance with standards, non-standard operations and maintenance, and shortage of skilled personnel as the most significant causes of boiler explosions. Hossan et al. [41] also utilized descriptive statistics to determine the causes of boiler accidents. They found ignorance of officials, lack of awareness, and lack of proper facilities to be the most critical causes of boiler accidents. In contrast to those studies, this research found that the most prominent barriers to sustainable boiler operations are the absence of water treatment facilities, fossil fuel burning and GHG emissions, and excessive consumption of groundwater. Moreover, no previous research focused on prioritizing the barriers to sustainable boiler operations and exploring the causal relationships among them, particularly in the apparel industry of an emerging economy. Therefore, the outcomes of this study are quite distinctive.

### 5.1 Theoretical implications

This study provides several important theoretical insights, which are as follows:

- Significant barriers to sustainable boiler operations in the apparel industry have been identified in this study. This research structure can be followed later to explore the barriers to other operational sustainability in different sectors.
- The study utilized the fuzzy DEMATEL approach to evaluate the identified barriers. The presented fuzzy theory-based technique can minimize the amount of ambiguity or inaccuracy in the dataset. The sensitivity analysis performed in this study also confirms the stability of the obtained results. This can provide future researchers important perspective on fuzzy theory-based techniques.
- The study ranks the barriers and explores the causal relationships among the barriers with visual representation. This ranking and the causal relations can guide future researchers in the relevant area.

### 5.2 Managerial and policy implication

The study has several important managerial and policy implications. For instance, the study findings suggest that the apparel manufacturing industry managers should emphasize developing preventative actions and measures to mitigate the boiler operation’s negative impacts and improve operational reliability and sustainability. Energy-efficient technology needs to be applied systematically in boiler operations, and developing a safe and hazard-free workplace for the workers is important as well to achieve sustainability.

The study’s findings point to some mitigation strategies for some significant barriers as well. For instance, one potential solution to overcome the "absence of water treatment facilities" in implementing sustainability in boiler operations is to use alternative water sources that do not require treatment, such as rainwater. Additionally, using closed-loop systems, where the water is recycled and reused within the boiler system, can also help reduce the need for water treatment. Again, proper blowdown control can minimize the total dissolved solids (TDS) in the boiler water. Regular monitoring and maintenance of the boiler water chemistry are also essential to ensure optimal boiler performance and sustainability.

To overcome issues like "fossil fuel burning and greenhouse gas (GHG) emissions", the industry can switch to renewable energy sources such as biomass, solar, or geothermal. The efficiency and emission reduction of traditional power plants can be improved by combining heat and power (CHP) systems that collect and use the heat that is often lost during the production of electricity. Improving boiler efficiency through regular maintenance, updating equipment, and using modern combustion processes can assist to minimise fuel use and emissions. The carbon dioxide (CO_2_) released by the boiler may be captured and stored underground using carbon capture and storage (CCS) technology. Emissions may be reduced with the aid of an energy management system that keeps tabs on the boiler’s power usage and adjusts settings as needed.

To overcome the issue of "excessive consumption of groundwater", alternative water sources such as rainwater or treated greywater can be used. These can be achieved by installing rainwater harvesting systems or greywater treatment systems. Another option is to use surface water sources like rivers, lakes, or streams instead of groundwater.

The policymakers can also obtain important insights from this study’s findings. For instance, the findings suggest implementing a robust compliance program helps the industry to operate efficiently within the legal bounds and reduces the likelihood of regulatory violations. Besides, regular internal audits and self-inspections can help identify potential issues and provide timely corrective action. Policymakers can use these insights to formulate long-term strategic plans to promote sustainability in various manufacturing industries.

### 5.3 Implications of achieving SDGs

This study can help the decision-makers of the relevant sector to contribute to attaining several specific SDGs. For instance, through sustainable practices in boiler operation, air pollution caused by the boiler can be reduced, leading to improved air quality and, subsequently, better health outcomes for individuals living in the area, which is closely related to SDG-3 (good health and well-being). Additionally, regular maintenance, training of personnel, and adherence to proper operating procedures can help reduce the risk of accidents and promote a safer working environment. Furthermore, sustainable practices in boiler operation can also contribute to SDG-3 by reducing the strain on healthcare systems and improving overall health and well-being.

Through sustainable practices, water contamination risk from boiler operation can be reduced by implementing proper treatment and discharge systems. Hence, sustainable boiler operations can conserve water resources by introducing treatment facilities and reducing water consumption, which can contribute to SDG-6 (safe and clean water).

The boiler’s energy efficiency can be increased by implementing sustainable practices in boiler operation, such as using cleaner fuels, implementing energy-efficient technologies, and regular maintenance. These can lead to reduced energy consumption, lower energy costs, and decreased greenhouse gas emissions. Furthermore, sustainable boiler operation can help promote renewable energy sources, such as solar or geothermal energy, which reduces the dependence on fossil fuels and the associated environmental impacts. All these can contribute to SDG-7 (affordable, sustainable and clean energy) and SDG-12 (sustainable management and efficient use of natural resources).

Promoting sustainable practices in boiler operation can encourage innovation in sustainable boiler technology and infrastructure. Investing in sustainable boiler operations can provide jobs and economic growth, which is essential for developing industries, and promoting innovations. All these can contribute to SDG-9 (industry, innovation, and infrastructure).

Furthermore, sustainable boiler operations can help to reduce GHG emissions and promote the use of renewable energy sources and carbon capture and storage technologies, which can help to reduce the amount of carbon dioxide in the atmosphere and thus mitigate the effects of climate change. These can contribute to SDG-13 (reduce the impacts of change).

## 6 Conclusions

Boiler operations are essential in the apparel manufacturing process. As a substantial part of apparel manufacturing and a significant consumer of natural resources, the sustainability of boiler operations must be ensured to attain various important SDGs. Workplace safety in the apparel industry is also highly dependent on sustainable boiler operations. Several barriers existing in the apparel industries of emerging economies create hindrances to attaining sustainable operations of boilers. This research is one of the first attempts in the literature to evaluate the barriers to sustainable boiler operation in an emerging economy like Bangladesh, a major global apparel manufacturer. A fuzzy DEMATEL-based framework was utilized in this study to reduce the vagueness and uncertainty of the human decision-making process. This study’s findings suggest that the ’Absence of water treatment facilities (B5)’, ’Fossil fuel burning and GHG emissions (B3)’, ’Excessive consumption of groundwater (B9)’, ’Inadequate compliance with safety and hazard regulations (B11),’, and ’Not recycling blowdown water (B8) as the top 5 most significant barriers. An interesting observation from this study is that ’Inadequate compliance with safety and hazard regulations (B11)’ and ’Lack of periodic inspection and preventive maintenance (B6)’ are the top 2 most important causal barriers, which indicates visibilities are not always the best indicator of influencing capability of a barrier. Managers and policymakers should utilize this insight to make more far-sighted and proactive strategies in the future.

Like all studies, this study also has some limitations that need to be addressed in future research. Although fuzzy DEMATEL is a highly effective tool for decision-making problems, it can be very complex with increasing the number of variables. This study dealt with 13 variables. Therefore each expert needed to respond to (13×12 = 156) semi-structured questions to form the direct-relation matrix, which is difficult for a human decision-maker. Other more flexible decision-making methods can be considered in future research attempts. In the future, researchers can also consider involving artificial intelligence and machine learning techniques to maintain decision-making consistency, which can make the study more robust. Future researchers can also consider other industrial operations to be analyzed using this framework to promote sustainability.

## Supporting information

S1 File (DOCX)
