# Supplementary material for: Evaluating barriers to sustainable boiler operation in the apparel manufacturing industry: Implications for mitigating operational hazards in the emerging economies
Source: PLoS One. 2023 Apr 14;18(4):e0284423. doi: 10.1371/journal.pone.0284423 (PMC10104351; doi:10.1371/journal.pone.0284423)
Supplement: S1 File — (DOCX) [file pone.0284423.s001.docx]

**Supporting information file**

- **Questionnaire for determining the relevant barriers**

*Q.1: What position do you hold in the boiler operations of the apparel industry?*

*Q.2: How many years of expertise do you have in Bangladesh's apparel industry?*

*Q.3: Please choose the key barriers to sustainable boiler operations in the apparel industry from the barriers provided below. Please respond 'Yes' if a barrier is critical to the adoption of sustainability in boiler operations of this sector in Bangladesh; else, write 'No'. You can also include other barriers critical for sustainable boiler operations at the end of the provided list.*

| **Barriers** | **Put "Yes" for relevant & "No" for irrelevant** |
| --- | --- |
| Usage of old and inefficient infrastructure |  |
| High dependency on nonrenewable energy sources |  |
| Fossil fuel burning and GHG emissions |  |
| Improper heat insulation |  |
| Absence of water treatment facilities |  |
| Lack of periodic inspection and preventive maintenance |  |
| Absence of heat recovery system |  |
| Lack of automation |  |
| Not recycling blowdown water |  |
| Excessive consumption of groundwater |  |
| Lack of skilled manpower |  |
| Inadequate compliance with safety and hazard regulations |  |
| Inadequate boiler room ventilation and safety measures |  |
|  | |
| **Please suggest new relevant barrier(s), if any** | |
| 1. | |
| 2. | |
| 3. | |
| 4. | |
| 5. | |

**Table S1:** Finally selected 13 barriers with brief descriptions

| **Serial No** | **Barriers** | **Description** |
| --- | --- | --- |
| 1 | Usage of old and inefficient infrastructure | An inefficient old boiler system uses more fuel and produces more carbon emissions than a modern, high-efficiency boiler. These old and inefficient infrastructures and systems not only contribute to the negative environmental impact but also increase operating costs. |
| 2 | High dependency on nonrenewable energy sources | The high dependence on nonrenewable energy sources can negatively impact the operation of a sustainable boiler. Additionally, it can lead to higher energy costs and potentially increased maintenance needs. |
| 3 | Fossil fuel burning and GHG emissions | Fossil fuel burning in boilers results in the emission of greenhouse gases (GHGs), which can negatively impact the operational sustainability of a boiler. The emissions contribute to climate change and air pollution, negatively affecting human health. |
| 4 | Improper heat insulation | Heat can escape from the boiler without proper insulation, leading to increased energy consumption and costs. Additionally, an inadequately insulated boiler may be unable to reach and maintain optimal operating temperatures, leading to reduced efficiency and increased wear and tear on the equipment. Furthermore, poor insulation leads to higher thermal losses, resulting in increased pollution and consumption of fossil fuels. |
| 5 | Absence of water treatment facilities | Without adequate water treatment, boilers may suffer from corrosion and mineral buildup, which can lead to reduced efficiency, increased maintenance needs, and shorter equipment lifespan. Additionally, untreated water can cause damage to the boiler's internal components and lead to unexpected shutdowns. Furthermore, the presence of impurities in the water might produce harmful by-products, such as sludge and scale, which can create a health hazard in the workplace. |
| 6 | Lack of periodic inspection and preventive maintenance | Small issues can go undetected without regular inspection and maintenance and grow into larger, more costly problems. Additionally, poorly maintained boilers may experience reduced efficiency, leading to increased energy consumption and higher operating costs. Furthermore, the lack of maintenance could cause unexpected shutdowns and an increase in downtime, which can greatly impact the production process and, in some cases, damage the equipment. |
| 7 | Absence of heat recovery system | A heat recovery system captures and reuses heat that would otherwise be wasted, increasing the overall energy efficiency of the boiler. Without a heat recovery system, a significant amount of energy is lost, resulting in increased energy consumption, higher operating costs, and increased greenhouse gas emissions. |
| 8 | Not recycling blowdown water | Blowdown water is removed from the boiler to control the level of dissolved solids in the water. If this water is not recycled, it results in a waste of water resources and increases water treatment costs, and can lead to the discharge of pollutants into the environment. |
| 9 | Excessive consumption of groundwater | If the underground water source in the boiler area becomes depleted, the facility may have to close temporarily or switch to an alternative water source. Additionally, over-pumping of groundwater can lead to subsidence, where the land surface sinks (generation of sinkhole) due to the underground water being pumped out faster than it can be replenished. These can damage the infrastructure of the facility, including the boiler. |
| 10 | Lack of skilled manpower | If the facility does not have enough trained personnel to operate and maintain the boiler, it may not be able to run at full capacity or may require frequent repairs. These can lead to increased costs and decreased efficiency. Additionally, a lack of skilled manpower may lead to improper boiler operation, resulting in safety hazards, equipment failure, and increased emissions. Furthermore, qualified personnel must conduct regular maintenance and inspection to address severe issues resulting in equipment failure. This maintenance is hampered if there is a shortage of skilled manpower. |
| 11 | Inadequate compliance with safety and hazard regulations | A boiler must be operated within the allowable safe and legal parameters. Doing otherwise may result in equipment failure or even an accident. These could cause problems like corrosion, leaks, and other damage that could lead to decreased efficiency and repair costs. Furthermore, non-compliance could also result in penalties, fines, or even the facility's shutdown. |
| 12 | Inadequate boiler room ventilation and safety measures | Lack of proper ventilation can lead to the buildup of dangerous gases such as carbon monoxide and nitrogen dioxide. These can be dangerous to personnel and equipment and cause equipment failures or even accidents. Additionally, poor ventilation can lead to the building up of excessive heat and humidity in the boiler room, which can cause corrosion and damage to the boiler and other equipment, reducing their lifespan and efficiency. |
| 13 | Inadequate supply and availability of spare parts | If a facility relies heavily on imported parts for the boiler, it may be at risk of supply disruptions or price fluctuations of components due to the volatility of the international market. This can lead to increased costs or even shutdowns. Additionally, if any specific supplier country has a restriction on imports due to geo-political or economic reasons, this could affect the regular operation of the facility as well. |

- **Fuzzy set theory**

Fuzzy logic deals with approximate reasoning rather than precise values and can handle the uncertainty of human judgment (Bari et al., 2022), which is the basis of the fuzzy set theory. Converting linguistic expressions into fuzzy values is preferable to just integrating judgments, ideas, or conclusions derived by individuals based on their collective knowledge.

Let, Ã $(a_{\mathrm{ij}})$ be a fuzzy judgment expressed as equation (1).

$a_{\mathrm{ij}}=\left( l_{\mathrm{ij}},m_{\mathrm{ij}},u_{\mathrm{ij}} \right)$ (1)

Here, $a_{\mathrm{ji}}=a_{\mathrm{ij}}{}^{-1}$ and all $a_{\mathrm{ij}}$ are triangular fuzzy numbers (TFN). The TFN is a well-known fuzzy number type, among several other types. A TFN, $a_{\mathrm{ij}}=\left( l_{\mathrm{ij}},m_{\mathrm{ij}},u_{\mathrm{ij}} \right)$ is with a lower point, $l_{\mathrm{ij}}$ and an upper point,$u_{\mathrm{ij}}$. Here, $m_{\mathrm{ij}}$ is the point where$\mu\left( x \right)=1$, which is a crisp value. $\mu\left( x \right)$ is the membership function of TFN. This membership function $\mu(x)$ of TFN (Bari et al., 2022) is shown in equation (2).

$\mu(x)=\left\{ \begin{matrix} \frac{x-l}{m-l}, & x\epsilon[l,m] \\ \frac{u-x}{u-m}, & x\epsilon[m,u] \\ 0, & \text{ otherwise } \end{matrix} \right.$ (2)

Where $l_{ij}\leq m_{\mathrm{ij}}\leq u_{\mathrm{ij}}.$ If $l_{\mathrm{ij}}=m_{\mathrm{ij}}=u_{\mathrm{ij}}$, the fuzzy number becomes a crisp number.

Defuzzification is an essential operation in fuzzy calculation by which a fuzzy number converts into a crisp number (Chakraverty et al., 2019). The centroid formula for defuzzification of the fuzzy number, $M_{1}=\left( l_{1},m_{1},u_{1} \right)$ is shown in equation (3).

$Defuzzified crisp number=\frac{u_{1}+2m_{1}+l_{1}}{4}$ (3)

- **The steps of the fuzzy-based DEMATEL framework**

*Step 1:* Gather linguistic feedback from each expert to determine one barrier's influence over another. The linguistic scale shown in **Table S2** is used to collect experts' feedback.

**Table S2:** Linguistic scale and corresponding fuzzy values

| **Linguistic terms** | **Corresponding Fuzzy values** |
| --- | --- |
| No influence (N) | [0, 0, 0] |
| Very Low influence (VL) | [0, 1, 2] |
| Low influence (L) | [1, 2, 3] |
| Medium influence (M) | [2, 3, 4] |
| High influence (H) | [3, 4, 5] |
| Very High influence (VH) | [5, 5, 5] |

*Step 2:* Convert the linguistic feedback into fuzzy feedback using **Table S2**. Linguistic opinions gathered from 10 experts were fuzzified to form 10 fuzzified direct-relation matrices.

*Step 3:* Form a fuzzy direct-relation matrix for each of the experts, thus forming 10 direct-relation matrices for 10 experts. The fuzzy direct-relation matrices are as equation (4).

$Ã_{n\times n}=\begin{matrix} B1 \\ B2 \\ B3 \\ \vdots\\ Bn \end{matrix}\left[ \begin{matrix} \left( 0,0,0 \right) & a_{12} & a_{13} & \cdots& a_{1n} \\ a_{21} & \left( 0,0,0 \right) & a_{22} & \cdots& a_{2n} \\ a_{31} & a_{32} & \left( 0,0,0 \right) & \cdots& a_{3n} \\ \vdots& \vdots& \vdots& \vdots& \vdots\\ a_{n1} & a_{n2} & a_{n3} & \cdots& \left( 0,0,0 \right) \end{matrix} \right]$ (4)

Here, $B1$ to $Bn$ are the barriers to be analyzed in this study.

*Step 4:* Aggregate all the direct-relation matrices into an aggregated direct-relation matrix by the arithmetic mean method. The aggregated direct-relationship matrix is presented in **Table S3**.

**Table S3**: Average fuzzy direct-relation matrix

|  | **B1** | | | **B2** | | | **B3** | | | **B4** | | | **B5** | | | **B6** | | | **B7** | | | **B8** | | | **B9** | | | **B10** | | | **B11** | | | **B12** | | | **B13** | | |
| --- | --- | --- | --- | --- | --- | --- | --- | --- | --- | --- | --- | --- | --- | --- | --- | --- | --- | --- | --- | --- | --- | --- | --- | --- | --- | --- | --- | --- | --- | --- | --- | --- | --- | --- | --- | --- | --- | --- | --- |
| **B1** | 0.00 | 0.00 | 0.00 | 0.00 | 0.00 | 0.00 | 2.00 | 3.00 | 4.00 | 0.25 | 0.67 | 1.08 | 0.00 | 0.25 | 0.50 | 0.00 | 0.83 | 1.67 | 0.17 | 0.33 | 0.50 | 0.00 | 0.00 | 0.00 | 0.25 | 1.17 | 2.08 | 0.25 | 0.58 | 0.92 | 0.00 | 0.00 | 0.00 | 0.00 | 0.17 | 0.33 | 0.00 | 0.17 | 0.33 |
| **B2** | 0.00 | 0.00 | 0.00 | 0.00 | 0.00 | 0.00 | 4.00 | 4.50 | 5.00 | 0.00 | 0.00 | 0.00 | 0.00 | 0.00 | 0.00 | 0.00 | 0.00 | 0.00 | 0.00 | 0.00 | 0.00 | 0.00 | 0.00 | 0.00 | 0.00 | 0.00 | 0.00 | 0.50 | 1.25 | 2.00 | 0.00 | 0.00 | 0.00 | 0.00 | 0.00 | 0.00 | 0.17 | 0.33 | 0.50 |
| **B3** | 0.00 | 0.00 | 0.00 | 0.17 | 1.17 | 2.17 | 0.00 | 0.00 | 0.00 | 0.00 | 0.00 | 0.00 | 0.00 | 0.00 | 0.00 | 0.00 | 0.00 | 0.00 | 0.00 | 0.00 | 0.00 | 0.00 | 0.00 | 0.00 | 0.00 | 0.00 | 0.00 | 0.00 | 0.00 | 0.00 | 0.00 | 0.00 | 0.00 | 0.00 | 0.00 | 0.00 | 0.08 | 1.00 | 1.92 |
| **B4** | 0.00 | 0.00 | 0.00 | 0.00 | 0.00 | 0.00 | 0.17 | 1.17 | 2.17 | 0.00 | 0.00 | 0.00 | 0.00 | 0.00 | 0.00 | 0.00 | 0.00 | 0.00 | 0.00 | 0.17 | 0.33 | 0.00 | 0.00 | 0.00 | 0.00 | 0.00 | 0.00 | 0.00 | 0.00 | 0.00 | 0.00 | 0.00 | 0.00 | 0.00 | 0.00 | 0.00 | 0.00 | 0.00 | 0.00 |
| **B5** | 0.00 | 0.42 | 0.83 | 0.00 | 0.00 | 0.00 | 0.58 | 1.58 | 2.58 | 0.00 | 0.00 | 0.00 | 0.00 | 0.00 | 0.00 | 0.00 | 0.83 | 1.67 | 0.00 | 0.17 | 0.33 | 2.17 | 3.17 | 4.17 | 4.00 | 4.50 | 5.00 | 0.00 | 0.42 | 0.83 | 0.00 | 0.00 | 0.00 | 0.00 | 0.00 | 0.00 | 0.00 | 0.33 | 0.67 |
| **B6** | 1.08 | 2.08 | 3.08 | 0.00 | 0.00 | 0.00 | 0.42 | 1.42 | 2.42 | 0.83 | 1.83 | 2.83 | 1.67 | 2.67 | 3.67 | 0.00 | 0.00 | 0.00 | 0.25 | 0.50 | 0.75 | 0.00 | 0.25 | 0.50 | 2.25 | 3.25 | 4.25 | 0.92 | 1.75 | 2.58 | 0.00 | 0.00 | 0.00 | 0.58 | 1.58 | 2.58 | 0.17 | 0.75 | 1.33 |
| **B7** | 0.00 | 0.00 | 0.00 | 0.00 | 0.00 | 0.00 | 2.00 | 3.00 | 4.00 | 0.00 | 0.17 | 0.33 | 0.00 | 0.00 | 0.00 | 0.00 | 0.17 | 0.33 | 0.00 | 0.00 | 0.00 | 0.00 | 0.83 | 1.67 | 0.00 | 0.25 | 0.50 | 0.00 | 0.00 | 0.00 | 0.00 | 0.00 | 0.00 | 0.00 | 0.17 | 0.33 | 0.00 | 0.17 | 0.33 |
| **B8** | 0.00 | 0.00 | 0.00 | 0.00 | 0.00 | 0.00 | 0.33 | 1.33 | 2.33 | 0.00 | 0.00 | 0.00 | 0.25 | 0.75 | 1.25 | 0.00 | 0.00 | 0.00 | 0.00 | 0.33 | 0.67 | 0.00 | 0.00 | 0.00 | 3.08 | 3.92 | 4.75 | 0.00 | 0.00 | 0.00 | 0.00 | 0.00 | 0.00 | 0.00 | 0.00 | 0.00 | 0.00 | 0.00 | 0.00 |
| **B9** | 0.00 | 0.25 | 0.50 | 0.00 | 0.00 | 0.00 | 0.42 | 0.83 | 1.25 | 0.00 | 0.00 | 0.00 | 0.00 | 0.58 | 1.17 | 0.00 | 0.00 | 0.00 | 0.00 | 0.00 | 0.00 | 0.00 | 1.00 | 2.00 | 0.00 | 0.00 | 0.00 | 0.00 | 0.00 | 0.00 | 0.00 | 0.00 | 0.00 | 0.00 | 0.00 | 0.00 | 0.00 | 0.08 | 0.17 |
| **B10** | 0.00 | 0.67 | 1.33 | 0.00 | 0.00 | 0.00 | 0.00 | 0.33 | 0.67 | 0.00 | 0.58 | 1.17 | 0.00 | 0.17 | 0.33 | 0.17 | 0.92 | 1.67 | 0.17 | 0.33 | 0.50 | 0.00 | 0.17 | 0.33 | 0.00 | 0.42 | 0.83 | 0.00 | 0.00 | 0.00 | 0.42 | 0.83 | 1.25 | 0.17 | 0.50 | 0.83 | 1.00 | 1.83 | 2.67 |
| **B11** | 1.83 | 2.83 | 3.83 | 0.00 | 0.00 | 0.00 | 0.33 | 1.08 | 1.83 | 3.00 | 4.00 | 5.00 | 5.00 | 5.00 | 5.00 | 2.42 | 3.00 | 3.58 | 1.92 | 2.92 | 3.92 | 0.42 | 0.83 | 1.25 | 3.25 | 4.08 | 4.92 | 1.33 | 2.33 | 3.33 | 0.00 | 0.00 | 0.00 | 2.17 | 2.75 | 3.33 | 0.00 | 0.00 | 0.00 |
| **B12** | 0.00 | 0.00 | 0.00 | 0.00 | 0.00 | 0.00 | 1.00 | 2.00 | 3.00 | 0.00 | 0.00 | 0.00 | 0.00 | 0.00 | 0.00 | 0.33 | 0.67 | 1.00 | 0.00 | 0.00 | 0.00 | 0.00 | 0.00 | 0.00 | 0.00 | 0.00 | 0.00 | 0.25 | 0.67 | 1.08 | 0.00 | 0.00 | 0.00 | 0.00 | 0.00 | 0.00 | 0.00 | 0.00 | 0.00 |
| **B13** | 1.42 | 2.42 | 3.42 | 0.17 | 0.58 | 1.00 | 0.00 | 0.17 | 0.33 | 0.00 | 0.00 | 0.00 | 2.00 | 3.00 | 4.00 | 0.33 | 0.67 | 1.00 | 0.33 | 0.67 | 1.00 | 0.17 | 0.33 | 0.50 | 0.25 | 1.25 | 2.25 | 1.92 | 2.75 | 3.58 | 0.00 | 0.00 | 0.00 | 0.00 | 0.00 | 0.00 | 0.00 | 0.00 | 0.00 |

*Step 5:* Determine the crisp direct-relation matrix by defuzzification of the fuzzy values. The aggregated fuzzified direct-relation matrix was converted into the crisp direct-relation matrix by the de-fuzzified operation mentioned in equation (3). The de-fuzzified crisp direct-relation matrix is provided in **Table S4**.

**Table S4:** Crisp relation matrix

|  | **B1** | **B2** | **B3** | **B4** | **B5** | **B6** | **B7** | **B8** | **B9** | **B10** | **B11** | **B12** | **B13** |
| --- | --- | --- | --- | --- | --- | --- | --- | --- | --- | --- | --- | --- | --- |
| **B1** | 0.00 | 0.00 | 3.00 | 0.67 | 0.25 | 0.83 | 0.33 | 0.00 | 1.17 | 0.58 | 0.00 | 0.17 | 0.17 |
| **B2** | 0.00 | 0.00 | 4.50 | 0.00 | 0.00 | 0.00 | 0.00 | 0.00 | 0.00 | 1.25 | 0.00 | 0.00 | 0.33 |
| **B3** | 0.00 | 1.17 | 0.00 | 0.00 | 0.00 | 0.00 | 0.00 | 0.00 | 0.00 | 0.00 | 0.00 | 0.00 | 1.00 |
| **B4** | 0.00 | 0.00 | 1.17 | 0.00 | 0.00 | 0.00 | 0.17 | 0.00 | 0.00 | 0.00 | 0.00 | 0.00 | 0.00 |
| **B5** | 0.42 | 0.00 | 1.58 | 0.00 | 0.00 | 0.83 | 0.17 | 3.17 | 4.50 | 0.42 | 0.00 | 0.00 | 0.33 |
| **B6** | 2.08 | 0.00 | 1.42 | 1.83 | 2.67 | 0.00 | 0.50 | 0.25 | 3.25 | 1.75 | 0.00 | 1.58 | 0.75 |
| **B7** | 0.00 | 0.00 | 3.00 | 0.17 | 0.00 | 0.17 | 0.00 | 0.83 | 0.25 | 0.00 | 0.00 | 0.17 | 0.17 |
| **B8** | 0.00 | 0.00 | 1.33 | 0.00 | 0.75 | 0.00 | 0.33 | 0.00 | 3.92 | 0.00 | 0.00 | 0.00 | 0.00 |
| **B9** | 0.25 | 0.00 | 0.83 | 0.00 | 0.58 | 0.00 | 0.00 | 1.00 | 0.00 | 0.00 | 0.00 | 0.00 | 0.08 |
| **B10** | 0.67 | 0.00 | 0.33 | 0.58 | 0.17 | 0.92 | 0.33 | 0.17 | 0.42 | 0.00 | 0.83 | 0.50 | 1.83 |
| **B11** | 2.83 | 0.00 | 1.08 | 4.00 | 5.00 | 3.00 | 2.92 | 0.83 | 4.08 | 2.33 | 0.00 | 2.75 | 0.00 |
| **B12** | 0.00 | 0.00 | 2.00 | 0.00 | 0.00 | 0.67 | 0.00 | 0.00 | 0.00 | 0.67 | 0.00 | 0.00 | 0.00 |
| **B13** | 2.42 | 0.58 | 0.17 | 0.00 | 3.00 | 0.67 | 0.67 | 0.33 | 1.25 | 2.75 | 0.00 | 0.00 | 0.00 |

*Step 6:* Compute the normalized direct crisp-relation matrix, $"X"$, by using equations (5) and (6).

$L=\frac{1}{{max}_{1\leq i\leq n}\sum_{j=1}^{n} a_{ij}}$ (5)

$X=L\times A$ (6)

Where *L* is a normalization factor, and *A* is a crisp-relation matrix. The normalized crisp relation matrix formed using equation (5) and equation (6) is provided in **Table S5**.

**Table S5**: Normalized direct crisp relation matrix

|  | **B1** | **B2** | **B3** | **B4** | **B5** | **B6** | **B7** | **B8** | **B9** | **B10** | **B11** | **B12** | **B13** |
| --- | --- | --- | --- | --- | --- | --- | --- | --- | --- | --- | --- | --- | --- |
| **B1** | 0.000 | 0.000 | 0.083 | 0.083 | 0.000 | 0.042 | 0.000 | 0.000 | 0.083 | 0.000 | 0.000 | 0.000 | 0.000 |
| **B2** | 0.000 | 0.000 | 0.208 | 0.000 | 0.000 | 0.000 | 0.000 | 0.000 | 0.000 | 0.083 | 0.000 | 0.000 | 0.000 |
| **B3** | 0.000 | 0.042 | 0.000 | 0.000 | 0.000 | 0.000 | 0.000 | 0.000 | 0.000 | 0.000 | 0.000 | 0.000 | 0.042 |
| **B4** | 0.000 | 0.000 | 0.042 | 0.000 | 0.000 | 0.000 | 0.000 | 0.000 | 0.000 | 0.000 | 0.000 | 0.000 | 0.000 |
| **B5** | 0.042 | 0.000 | 0.125 | 0.000 | 0.000 | 0.042 | 0.000 | 0.167 | 0.167 | 0.042 | 0.000 | 0.000 | 0.000 |
| **B6** | 0.083 | 0.000 | 0.083 | 0.042 | 0.125 | 0.000 | 0.000 | 0.000 | 0.125 | 0.125 | 0.000 | 0.042 | 0.042 |
| **B7** | 0.000 | 0.000 | 0.125 | 0.000 | 0.000 | 0.000 | 0.000 | 0.000 | 0.000 | 0.000 | 0.000 | 0.000 | 0.000 |
| **B8** | 0.000 | 0.000 | 0.083 | 0.000 | 0.125 | 0.000 | 0.000 | 0.000 | 0.167 | 0.000 | 0.000 | 0.000 | 0.000 |
| **B9** | 0.042 | 0.000 | 0.083 | 0.000 | 0.042 | 0.000 | 0.000 | 0.042 | 0.000 | 0.000 | 0.000 | 0.000 | 0.000 |
| **B10** | 0.042 | 0.000 | 0.042 | 0.000 | 0.042 | 0.000 | 0.000 | 0.042 | 0.042 | 0.000 | 0.000 | 0.000 | 0.083 |
| **B11** | 0.125 | 0.000 | 0.042 | 0.167 | 0.208 | 0.000 | 0.125 | 0.083 | 0.167 | 0.083 | 0.000 | 0.000 | 0.000 |
| **B12** | 0.000 | 0.000 | 0.083 | 0.000 | 0.000 | 0.000 | 0.000 | 0.000 | 0.000 | 0.083 | 0.000 | 0.000 | 0.000 |
| **B13** | 0.083 | 0.000 | 0.042 | 0.000 | 0.125 | 0.000 | 0.000 | 0.083 | 0.083 | 0.042 | 0.000 | 0.000 | 0.000 |

*Step 7:* Determine the total-relation matrix $"T"$ with equation (7).

$T=X\times{(I-X)}^{-1}$ (7)

Where *I* represent the identity matrix. The resulting total relation matrix $"T"$ is provided in **Table S6**.

**Table S6:** Total relation matrix

|  | **B1** | **B2** | **B3** | **B4** | **B5** | **B6** | **B7** | **B8** | **B9** | **B10** | **B11** | **B12** | **B13** |
| --- | --- | --- | --- | --- | --- | --- | --- | --- | --- | --- | --- | --- | --- |
| **B1** | 0.01 | 0.00 | 0.10 | 0.09 | 0.01 | 0.04 | 0.00 | 0.01 | 0.09 | 0.01 | 0.00 | 0.00 | 0.01 |
| **B2** | 0.01 | 0.01 | 0.22 | 0.00 | 0.01 | 0.00 | 0.00 | 0.01 | 0.01 | 0.09 | 0.00 | 0.00 | 0.02 |
| **B3** | 0.00 | 0.04 | 0.01 | 0.00 | 0.01 | 0.00 | 0.00 | 0.01 | 0.01 | 0.01 | 0.00 | 0.00 | 0.04 |
| **B4** | 0.00 | 0.00 | 0.04 | 0.00 | 0.00 | 0.00 | 0.00 | 0.00 | 0.00 | 0.00 | 0.00 | 0.00 | 0.00 |
| **B5** | 0.06 | 0.01 | 0.18 | 0.01 | 0.04 | 0.05 | 0.00 | 0.19 | 0.22 | 0.05 | 0.00 | 0.00 | 0.01 |
| **B6** | 0.11 | 0.01 | 0.15 | 0.05 | 0.15 | 0.01 | 0.00 | 0.04 | 0.18 | 0.14 | 0.00 | 0.04 | 0.06 |
| **B7** | 0.00 | 0.01 | 0.13 | 0.00 | 0.00 | 0.00 | 0.00 | 0.00 | 0.00 | 0.00 | 0.00 | 0.00 | 0.01 |
| **B8** | 0.02 | 0.01 | 0.12 | 0.00 | 0.14 | 0.01 | 0.00 | 0.03 | 0.20 | 0.01 | 0.00 | 0.00 | 0.01 |
| **B9** | 0.05 | 0.00 | 0.10 | 0.00 | 0.05 | 0.00 | 0.00 | 0.05 | 0.02 | 0.00 | 0.00 | 0.00 | 0.00 |
| **B10** | 0.06 | 0.00 | 0.07 | 0.00 | 0.06 | 0.01 | 0.00 | 0.06 | 0.08 | 0.01 | 0.00 | 0.00 | 0.09 |
| **B11** | 0.15 | 0.01 | 0.15 | 0.18 | 0.24 | 0.02 | 0.13 | 0.14 | 0.25 | 0.10 | 0.00 | 0.00 | 0.01 |
| **B12** | 0.00 | 0.00 | 0.09 | 0.00 | 0.01 | 0.00 | 0.00 | 0.01 | 0.01 | 0.08 | 0.00 | 0.00 | 0.01 |
| **B13** | 0.10 | 0.00 | 0.09 | 0.01 | 0.15 | 0.01 | 0.00 | 0.12 | 0.14 | 0.05 | 0.00 | 0.00 | 0.01 |

Note: Colored values are ≥ threshold value (**0.0957).**

*Step 8:* Compute the row (*D_i_*) and column (*R_j_*) sums from the total-relation matrix, T using equations (8) and (9).

$D_{i}=[\sum_{j=1}^{n} T_{ij}] \forall i$ (8)

$R_{i}=[\sum_{i=1}^{n} T_{ij}] \forall j$ (9)

*Step 9:* Compute the overall prominence (*P_i_*) and the net effect (*E_i_*) using equations (10) and (11).

$P_{i}=[D_{i}{+R}_{j}]\forall i=j$ (10)

$E_{i}=[D_{i}{-R}_{j}]\forall i=j$ (11)

The greater the value of *P_i_* for a barrier indicates greater prominence (i.e., the influence, importance, and visibility) of that barrier in terms of the overall relationship with other barriers. If *E_i_* > 0 for a barrier, then that barrier is causal; otherwise an *effect* barrier.

*Step 10:* Plot *P_i_* and *E_i_* values on a two-dimensional axis for each barrier to creating the cause-effect diagram. The influences of one barrier on other barriers can be depicted by the digraph obtained. To avoid comparably negligible effects, a threshold value ($\theta$) is set. If, $T_{ij}>\theta$ for a barrier B_i_, then that barrier influences or causes the barrier B_j_. A directed arrow is incorporated in the digraph to show this causal relation. Equation (12) is the mathematical expression for the calculation threshold value ($\theta)$.

$\theta= \mu+\sigma$ (12)

Here, *μ* is the mean value, and *σ* the standard deviation of the elements of the total-relation matrix, *T*. The threshold value was determined to be 0.0957, given that the matrix's mean was 0.0374 and its standard deviation was 0.0583.

**References:**

Bari, A. M., Siraj, M. T., Paul, S. K., & Khan, S. A. (2022). A hybrid multi-criteria decision-making approach for analyzing operational hazards in Heavy Fuel Oil-based power plants. *Decision Analytics Journal*, 100069.
